# The Role of mTOR Inhibitors in Liver Transplantation: Reviewing the Evidence

**DOI:** 10.1155/2014/845438

**Published:** 2014-02-25

**Authors:** Goran B. Klintmalm, Björn Nashan

**Affiliations:** ^1^Baylor Simmons Transplant Institute, Baylor University Medical Center, 3410 Worth Street, Suite 950, Dallas, TX 75246, USA; ^2^Department of Hepatobiliary Surgery and Visceral Transplantation, University Medical Center Eppendorf, Martinistra**β**e 52, 20246 Hamburg, Germany

## Abstract

Despite the success of liver transplantation, long-term complications remain, including *de novo* malignancies, metabolic syndrome, and the recurrence of hepatitis C virus (HCV) and hepatocellular carcinoma (HCC). The current mainstay of treatment, calcineurin inhibitors (CNIs), can also worsen posttransplant renal dysfunction, neurotoxicity, and diabetes. Clearly there is a need for better immunosuppressive agents that maintain similar rates of efficacy and renal function whilst minimizing adverse effects. The mammalian target of rapamycin (mTOR) inhibitors with a mechanism of action that is different from other immunosuppressive agents has the potential to address some of these issues. In this review we surveyed the literature for reports of the use of mTOR inhibitors in adult liver transplantation with respect to renal function, efficacy, safety, neurological symptoms, *de novo* tumors, and the recurrence of HCC and HCV. The results of our review indicate that mTOR inhibitors are associated with efficacy comparable to CNIs while having benefits on renal function in liver transplantation. We also consider newer dosing schedules that may limit side effects. Finally, we discuss evidence that mTOR inhibitors may have benefits in the oncology setting and in relation to HCV-related allograft fibrosis, metabolic syndrome, and neurotoxicity.

## 1. Introduction

One-year survival rates for liver transplantation currently stand at more than 80% in the US and Europe [1, 2]; however, the demand for liver transplants far outstrips the number of available donor livers as increasing numbers of patients are referred for transplantation. Moreover, the global incidence of conditions that may ultimately require a liver transplant (hepatocellular carcinoma (HCC), nonalcohol fatty liver disease, and cirrhosis) is predicted to increase [3–6], which would further drive demand for the procedure. This may be balanced by a reduction in liver transplants required owing to hepatitis C virus (HCV) as a result of the use of new potent antivirals. The success of liver transplantation is limited by shortages of suitable donor organs, adverse events of immunosuppressive drugs, and recurrence of disease.

Although transplant surgery was an area of great interest in the 1960s and 70s, the mortality rate for liver transplantation in 1978, using azathioprine and prednisone immunosuppression, was approximately 75%. Cyclosporine, a calcineurin inhibitor (CNI), changed the face of transplantation, and in a few years the survival rate of liver transplantation had reached 80% [7]. The search for new and safer immunosuppressants continued and in 1989, reports were published on the successful use of tacrolimus, another CNI, in liver transplantation [8]. CNIs have been the cornerstone of maintenance immunosuppression in liver transplantation [9], but their nephrotoxic effects are an important source of morbidity [10–13]. Several other factors are implicated in the development of renal dysfunction following liver transplantation, including increased age, diabetes mellitus, hypertension, and preexisting kidney disease [14]. Data from the United Network for Organ Sharing demonstrate that almost 20% of liver transplant recipients have chronic renal failure 5 years after transplantation [14]. High rates of renal dysfunction associated with the preexisting liver disease and with the use of CNIs are compounded by the use of the Model for End-Stage Liver Disease score for allocating transplants since it favors liver transplantation in individuals with renal dysfunction.

In addition to renal dysfunction, long-term complications associated with liver transplantation include the development of *de novo* malignancies and the recurrence of HCV and HCC. Recurrence of HCC occurs in approximately 20% of liver recipients [15] and is associated with poor prognosis [16]. In patients who receive a transplant due to HCV-related end-stage liver disease, graft reinfection is almost universal and a significant percentage of patients develop chronic hepatitis in the graft [17–19]; 5-year survival rates after primary liver transplantation are significantly reduced among HCV-positive patients compared to HCV-negative patients [19]. A four-fold greater risk of developing *de novo* malignancies posttransplant compared to the general population has also been reported [20].

Another concern relates to adverse effects of the immunosuppressants that are required to maintain the graft. For example, new-onset diabetes mellitus (NODM) has been estimated to occur in 5–27% of liver transplant recipients [21–23] and is associated with a negative impact on patient and graft survival [24]. CNIs, particularly tacrolimus, have been shown to increase the risk of developing NODM [21, 25, 26] and are also associated with an increase in the incidence of malignancies are transplantation [20, 27, 28] and with cases of neurotoxicity [28–30]. In addition, metabolic syndrome, which refers to the combination of abdominal obesity, hypertension, hyperglycemia, and hyperlipidemia, is common after liver transplantation and has been reported to affect 43–58% of liver transplant recipients [31]. Hypertension is also associated with CNIs, particularly with cyclosporine [32].

**Figure 1:**
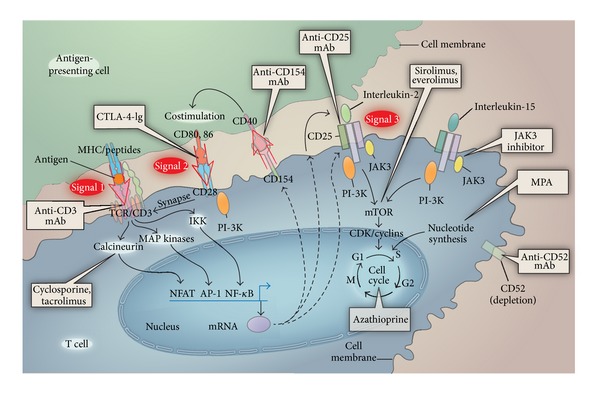
Sites of action of immunosuppressive drugs (adapted from [33] with permission).

Taken together, an unmet need clearly remains for identifying alternative immunosuppressive regimens that (1) maintain antirejection efficacy with substantially reduced CNI exposure; (2) optimize renal function, both short- and long-term, by minimizing CNI nephrotoxicity; (3) avoid or minimize CNI-associated adverse events; (4) reduce the recurrence of HCV and HCC; (5) reduce the occurrence of *de novo* posttransplant malignancies. The mammalian target of rapamycin (mTOR) inhibitors could potentially meet these criteria, in part because they allow the use of immunosuppressive regimens that include reduced doses of CNIs. The mTOR inhibitors also possess a mechanism of action that is different from other classes of immunosuppressants: sirolimus and everolimus engage FKBP12 to create complexes that engage and inhibit the target of rapamycin but cannot inhibit calcineurin (Figure 1). Inhibition of the target of rapamycin blocks signal 3 by preventing cytokine receptors from activating the cell cycle [33]. In addition, mTOR inhibitors may promote tolerance through actions on regulatory T-cells and dendritic cells [34, 35].

Two mTOR inhibitors are currently approved for use in transplantation. Everolimus is approved by the FDA for renal and liver transplantation and by the EMA for renal, heart and liver transplantation (Certican and Zortress, Novartis Pharma AG; Basel, Switzerland) [36, 37]. Clinical experience with everolimus in liver transplantation is limited by the fact that it was only recently approved for liver transplantation and it was not approved for renal transplantation in the EU until 2003 and in the US until 2010. Sirolimus is approved by the European Medicines Agency (EMA) and the US Food and Drug Administration (FDA) for renal transplantation (Rapamune, Pfizer, NY, USA) [38, 39]. In the US, sirolimus was approved for renal transplantation in 1999. Although not approved for liver transplantation, it has still been used in several centres in liver transplant recipients.

Everolimus is a derivative of sirolimus, differing by one extra hydroxyethyl group at position 40 [40]. In human studies, everolimus has a shorter half life (30 hours) compared to the 62 hours of sirolimus and a quicker time to steady state (4 days versus 6 days) [36, 38]. Both everolimus and sirolimus are substrates in the p-glycoprotein and cytochrome P450-3A4 pathways [40, 41]. Therefore, the absorption and clearance of mTOR inhibitors may be influenced by drugs that affect cytochrome P450-3A4 and/or p-glycoprotein, including common drugs such as fluconazole, azithromycin, and protease inhibitors [36, 38, 39]. An important interaction of mTOR inhibitors is with cyclosporine, with simultaneous administration leading to increases in blood levels of mTOR inhibitor [42, 43], although this appears to be more pronounced with sirolimus than with everolimus, necessitating the administration of sirolimus 4 hours after cyclosporine [38, 39].

Initial enthusiasm for the use of mTOR inhibitors in liver transplantation was tempered when the FDA issued a black box warning for *de novo* sirolimus use in liver transplantation after two studies reported hepatic artery thrombosis (HAT) [38, 44]. In 2009, the FDA issued a second black box warning, after a trial that compared conversion from CNIs to sirolimus versus continued CNI use showed that the number of deaths (3.8% (15/393) versus 1.4% (3/214)) was higher in the conversion group, although this was not significant. In addition, the rates of premature study discontinuation, overall adverse events (specifically infections), and biopsy-proven acute liver graft rejection at 12 months were all significantly higher in the conversion group compared to the group continuing with CNIs [38]. Unfortunately, it was not until recently that the complete data that led to these warnings were published [45] allowing them to be properly scrutinized [46]. Notwithstanding the warnings, the Scientific Registry of Transplant Recipients (SRTR) indicates that between 1999 and 2008 in the US, sirolimus and everolimus were used in 8.8% and 0.2% of liver transplant recipients, respectively, as maintenance therapy from the period of discharge to 1 year after transplantation [47]. Given the large amount of data available on the use of mTOR inhibitors in liver transplantation and the controversy surrounding the black box warnings, we have revisited the use of mTOR inhibitors in liver transplantation. To this end, we searched the literature from 2001 to 2012 to determine whether the clinical evidence supports a role for this class of immunosuppression with respect to efficacy, safety, and the ability to address unmet clinical needs.

## 2. Methods

### 2.1. Identification of Published Clinical Data regarding the Use of mTOR Inhibitors in Liver Transplantation

We searched the bibliographic database, PubMed, for studies published from January 2001 to April 2012. The following search criteria were used in the PubMed search: “everolimus liver transplantation” OR “sirolimus liver transplantation” OR “everolimus liver transplant” OR “sirolimus liver transplant.” Prospective or retrospective clinical studies and reviews of single transplantation centers were considered. We only included studies that met the following criteria: (1) focus on adult liver transplant recipients receiving immunosuppression with mTOR inhibitors, (2) publication in English, and (3) a patient sample size of at least *n* = 7 in the mTOR inhibitor treatment group. The studies identified were not subjected to a systematic review but are summarized and discussed based on the combined clinical experience of the authors. Dr Klintmalm has been involved in the use of mTOR inhibitors in over 1,500 patients at the Simmons Transplantation Institute at Baylor University Medical Center, including 650 liver transplant recipients converted to an mTOR inhibitor, 525 liver transplant recipients who received *de novo* therapy, and over 375 kidney transplant recipients. Professor Nashan has been using mTOR inhibitors (both *de novo* and conversion) in liver and renal transplantation for the last 17 years and in that time has been involved in numerous key clinical trials of mTOR inhibitors for both indications.

### 2.2. Identification of Pertinent Studies Presented at Recent Liver Transplant Congresses

We retrieved abstracts reporting results from trials pertaining to liver transplantation and everolimus or sirolimus that were presented at the following congresses: the American Transplant Congress, 2011; The 62nd and 63rd Annual Meetings of the American Association for the Study of Liver Diseases (AASLD), 2011 and 2012; 15th Congress of the European Society for Organ Transplantation, 2011; 23rd International Congress of the Transplantation Society, 2010; the 2011 Joint International Congress of the International Liver Transplantation Society (ILTS); the European Liver and Intestine Transplant Association; the Liver Intensive Care Group of Europe.

### 2.3. Identification of Ongoing Clinical Trials on the Use of mTOR Inhibitors in Liver Transplantation

The online database ClinicalTrials.gov was searched for ongoing clinical trials investigating mTOR inhibitors in liver transplantation.

### 2.4. Organization of Studies by Dosage Level and Dosing Strategy

We divided the studies according to their stated mode of mTOR inhibitor administration, that is, *de novo* (given from the time of transplantation) or conversion (where liver transplant recipients were converted to an mTOR inhibitor). In a small number of studies mTOR inhibitors were not given *de novo* but at some point after transplantation without the previous immunosuppression being stated; these are termed maintenance. We further classified the conversion studies according to whether sirolimus or everolimus was started “early” or “late,” because evidence suggests that the timepoint at which conversion takes place has an impact on renal function [10]. NODM and hypertension are also associated with the use of CNIs (see Introduction 1), so early conversion would also be expected to have a positive impact on these endpoints. We defined early conversion as administration of an mTOR inhibitor at 3 months or less after transplant and late conversion as conversion after 3 months after transplant, in line with other studies [48–50]. All studies were also classified according to a scoring system taking into account mTOR inhibitor dosage level and the strength of the study design. Dosage level was included in the scoring system since serious adverse events appear to be associated with higher doses of sirolimus and everolimus [44, 45, 51, 52]. We therefore based our dosage cut-off points on levels that are associated with less serious adverse events [46, 52]. For *de novo* and maintenance studies, the following criteria scored one point each: (a) inclusion of a control arm; (b) sirolimus or everolimus dose of ≤2 mg/day; and (c) serum levels of ≤10 ng/mL for sirolimus or ≤12 ng/mL for everolimus. In the case of conversion studies, the following criteria scored one point each: (a) a predefined conversion timepoint of ≤3 months after transplantation; (b) inclusion of a control arm; (c) specified dose of mTOR inhibitor (see above); and (d) specified serum level of mTOR inhibitor (see above). The studies were then classified as low (0-1 point), medium (2 points), or high (≥3 points) quality.

## 3. Results

Our literature search retrieved 40 studies on sirolimus use and 16 studies on everolimus use (Tables 1(a)–1(d)). We analyzed these studies according to renal function, efficacy, safety, metabolic syndrome, HCC, neurological symptoms, HCV recurrence/fibrosis progression, and *de novo *tumors.

### 3.1. Efficacy of mTOR Inhibitors

#### 3.1.1. Sirolimus

A retrospective study evaluating the use of *de novo *sirolimus versus CNIs found that patients who received sirolimus (*n* = 252) exhibited similar rates of patient and graft survival in comparison to liver transplant recipients receiving CNIs (*n* = 291). The percentage of patients who developed acute cellular rejection or those with BPAR was significantly lower in patients receiving sirolimus compared with those on CNIs (Table 2(a)) [53]. In a large retrospective study that included *de novo *sirolimus use in liver transplantation compared with a CNI control group there were no significant differences in rates of mortality or graft loss during the first year after liver transplant (Table 2(a)) [54].

In a high quality retrospective study assessing conversion (early versus late conversion), rejection rates in the sirolimus groups (early conversion: 35%; late conversion: 38%) were comparable to those in the CNI group (43%) [48]. In another retrospective study assessing conversion (medium quality), BPAR among patients converted at various times was 3.4% [67], while a medium quality review of a prospectively maintained database revealed an acute cellular rejection rate of 17.2% for those treated *de novo* with sirolimus versus 2.8% for those converted at various timepoints to sirolimus in response to rising serum creatinine concentrations [69].

Two single-arm prospective studies and one randomized study (Table 2(a)) have shown that late conversion to sirolimus in liver transplant recipients is associated with generally low rates of acute rejection: 4.8% [83], 6.7% [82] and 7.7% [78]. In addition, a high quality retrospective study demonstrated low rates of acute rejection: 5% among patients converted to sirolimus versus 4% for matched control group maintained on reduced-dose CNIs alone [72]. However, in a large, prospective randomized trial in which liver transplant recipients were converted late (more than 50% of patients in each group entered the study at least 3 years posttransplantation) and abruptly (within 24 hours) from CNI treatment to sirolimus, there were detrimental effects on efficacy and safety, suggesting that an overlap period is necessary [45]. The rate of BPAR (11.7% versus 6.1%) at 12 months after randomization (*P* = 0.02) and overall treatment failure (acute cellular rejection or discontinuation; 48.3% versus 26.7%; *P* < 0.001) was significantly higher in the sirolimus group compared to the control group who received CNI for up to 6 years. In addition, significantly more patients in the sirolimus group experienced ≥1 treatment-emergent adverse event during the study compared to the CNI group (*P* = 0.005) [45].

#### 3.1.2. Everolimus

In a study in which *de novo* liver transplant recipients were randomized to receive cyclosporine plus everolimus (*n* = 89) at either 1, 2 or 4 mg per day, BPAR rates were 32.1%, 26.7%, and 25.8%, respectively, versus 40% for the 30 patients receiving cyclosporine plus placebo, although this difference was not significant [52]. There was evidence of a dose relationship for treated acute rejection throughout the double-blind study period, with higher rates of acute rejection observed in patients on lower doses of everolimus (particularly the lowest dose of 1 mg/day). In this study, there were few deaths with patient survival reported as 83.3%, 82.1%, 96.7%, and 87.1% in liver transplant recipients who received placebo or everolimus at 1, 2, or 4 mg per day respectively; no deaths were considered to be treatment-related [52]. Rejection rates at 1 year after transplantation in a prospective, randomized study in which patients received either *de novo *everolimus or tacrolimus were 11% versus 3%, respectively [88]. In a high quality maintenance study in which liver transplant recipients were randomized at 1 month after transplant to either everolimus-facilitated elimination of tacrolimus, everolimus-facilitated reduction of tacrolimus, or standard-dose tacrolimus, withdrawing tacrolimus did not provide sufficient efficacy with a BPAR rate of 19.9% (although everolimus did allow substantial tacrolimus reduction in *de novo* liver transplant recipients while resulting in a significantly lower rate of BPAR at 1 year) [87].

In five early conversion studies (Table 2(b)), all of which were high quality, prospective, randomized trials, efficacy was either similar to [50, 89, 91] or better than [87, 90] control groups. One of these investigated whether everolimus could be used to withdraw or reduce immunosuppression with tacrolimus in *de novo *liver transplant recipients [87]. The results of this study showed that withdrawing tacrolimus did not provide sufficient efficacy with a BPAR rate of 19.9%. However, everolimus did allow substantial tacrolimus reduction in *de novo* liver transplant recipients while resulting in a significantly lower rate of BPAR at 1 year (4.1% versus 10.7%: everolimus + reduced-dose tacrolimus versus standard-dose tacrolimus, *P* = 0.005). The lower rate of BPAR was maintained at 24 months (6.1% versus 13.3%, *P* = 0.010) [90]. In a second study, at 1 year there were similar rates of patient survival (95.8% versus 95.9%), graft loss (2.1% versus 2.0%), and BPAR (17.7% versus 15.3%) in patients converting to everolimus versus those remaining on CNI treatment [50]. Similarly, in the extension phase of this study, at 35 months there were similar rates of patient survival (EVR: 95.7% versus CNI: 90.0%, *P* = 0.535), BPAR (24.4% versus 15.8%, *P* = 0.434), and efficacy failure (29.8% versus 28.2%, *P* = 0.903) [91].

In four late-conversion studies (two prospective and two retrospective; Table 2(b)), the incidence of BPAR up to 1 year after conversion was 1.6% [98], 2.8% [96], 9% [99] and 15% [95].

### 3.2. The Effect of mTOR Inhibitors on Renal Function 

#### 3.2.1. Sirolimus

In two large retrospective studies (one high quality and one low quality) in which patients received sirolimus as *de novo *therapy, there were reductions in the glomerular filtration rate (GFR) up to 1 year [54] or up to 5 years after transplant [55]. In contrast, in a third *de novo*, retrospective (low quality) study, modest improvements in renal function in patients receiving sirolimus were recorded at both 6 and 12 months after transplant, with creatinine levels decreasing by 0.22 and 0.28 mg/dL, respectively, compared to increases in creatinine of 0.61 and 0.35 mg/dL, respectively, in a control group receiving a standard immunosuppression regimen [15] (Table 3(a)).

Four retrospective studies (two high quality and two medium quality) reported renal function in liver transplant recipients converted early to sirolimus from CNI treatment (Table 3(b)) [48, 49, 67, 68]. Two of these studies included a control group. In the first retrospective study in which 72 liver transplant recipients converted to sirolimus from CNI treatment were stratified according to whether they had been converted <90 days from transplantation or after this period [48], there were significantly higher estimated GFR (eGFR) levels in patients converting at <90 days after transplant compared to those converting after day 90, at 3, 9, and 12 months after conversion. The CNI control group showed a significant decline in GFR at the last followup (last clinic visit date with laboratory value assessment) compared to pre-transplant [48]. In the second early-conversion study to include a control group [68], a cohort of 202 patients converted to sirolimus due to nephrotoxicity was compared with a control group of 876 patients who had not received sirolimus at any point after transplant; both groups had GFR <50 mL/min. There was a significant increase in measured GFR that persisted for 5 years after conversion in patients who converted to sirolimus at either 3 months or 1 year after transplant. As might be expected, conversion at 3 months produced greater improvements in 5-year renal function than conversion at 1 year (GFR: +24.3 cc/minute versus +16.3 cc/minute, resp.). In contrast, there was no difference in GFR after sirolimus conversion at 2 years after transplant, and later conversions at 5 years and 10 years after transplant resulted in a significantly decreased GFR [68]. In the two early-conversion studies that did not include a control group, significant improvements in GFR from baseline were observed in patients converting to sirolimus up to 1 year [49] and 3 years [67] after conversion.

Thirteen studies investigated the effect on renal function of late conversion to sirolimus in liver transplant recipients with impaired renal function related to the use of CNIs (Table 3(b)). Three prospective and four retrospective studies (a mixture of low- and medium quality) demonstrated improvements in renal function in recipients converting to sirolimus [77, 78, 81, 83–86]. Two of these studies (one prospective, one retrospective) demonstrated long periods of improved GFR after conversion in sirolimus conversion groups at 27.5 months [81] and up to 60 months after conversion [77]. Two small prospective studies (one low quality, single-arm and one medium quality, randomized) showed only numerical improvements at 6 [82] and 12 [76] months after conversion. In a third prospective, single-arm study of 28 liver transplant recipients, 14 were maintained on sirolimus and had stable renal function, while seven were unable to tolerate sirolimus and six progressed to end-stage renal disease [74]. One low quality prospective, randomized study [45] and two high quality retrospective studies failed to demonstrate significant improvements in renal function [71, 72].

From our literature search, proteinuria was observed in six liver transplant studies of variable quality involving sirolimus use (Table 3(d)) [67, 72, 73, 75, 77, 86]. In one of these, a small, prospective, randomized study, the rate of proteinuria during the 1-year followup was similar to that observed in controls receiving mycophenolate mofetil (MMF) [73]. However, in a retrospective early-conversion study, the incidence and severity of proteinuria increased following conversion, with rates of patients with moderate proteinuria (1–3 g/L) increasing from 14% (pre-conversion) to 27% (last followup at 5 years after conversion) and patients with severe (>3 g/L) proteinuria increasing from 7% (pre-conversion) to 11% (last followup) [67]. In addition, in a more recent retrospective study of 102 liver transplant recipients converted to sirolimus (due to nephrotoxicity associated with CNI use), after a median of 3.1 years, 24-hour urinary protein excretion increased from a median of 72 to 282 mg/day (*P* = 0.0001). Postsirolimus proteinuria ≥150 mg/day developed in 81% of patients after a median of 3.1 years of followup [86]. Independent predictors of massive proteinuria, defined as a peak urinary protein excretion ≥1000 mg/day, were a sirolimus trough level greater than 10 ng/mL, after transplant diabetes and lower eGFR (32.1 ± 10.6 mL/min versus 43.0 ± 17.5 mL/min, *P* = 0.004) at the time of sirolimus initiation [86].

#### 3.2.2. Everolimus

In a double-blind prospective randomized study (low quality) that administered *de novo* everolimus (*n* = 89) or placebo (*n* = 30) to liver transplant recipients receiving cyclosporine, there was no improvement in renal function, with liver transplant recipients receiving everolimus showing a decrease in creatinine clearance at 6 months after transplant (Table 2(c)) [52].

Four high quality, prospective, randomized studies showed good results in liver transplant recipients converted early to everolimus from CNI treatment (Table 3(c)) [50, 87, 89, 91]. One of these evaluated whether early CNI withdrawal and initiation of everolimus monotherapy in *de novo* liver transplantation patients would lead to superior renal function, compared to the cyclosporine control, at 12 months after transplantation [89]. At randomization, the mean eGFR value calculated by the modification of diet in renal disease (MDRD) formula was 81.7 ± 29.5 mL/min/1.73 m^2^ in the everolimus group and 74.7 ± 24.6 mL/min/1.73 m^2^ in the cyclosporine group (*P* = 0.30). At 6 and 12 months, respectively, the mean eGFR values in the everolimus group were 87.8 ± 36.7 and 87.6 ± 26.1 mL/min versus 58.2 ± 17.9 and 59.9 ± 12.6 mL/min in the cyclosporine group (*P* < 0.001 for both the 6- and 12-month comparisons). In a per-protocol analysis, the incidence of stage ≥3 chronic kidney disease (estimated GFR < 60 mL/min) was significantly lower in the everolimus group at 1 year after liver transplant (52.2% versus 15.4%, in the cyclosporine group, respectively, *P* = 0.005) [89]. More recently, results from an 11-month, multicenter, prospective, open-label trial were published in which liver transplant recipients with good renal function at 4 weeks after transplant were randomized to either continue CNI treatment with/without corticosteroids (*n* = 102) or switch to everolimus with/without corticosteroids (*n* = 101) [50]. There was a significant difference between treatments using the MDRD formula (−7.8 mL/min in favor of everolimus, *P* = 0.021), although this was not significant when using the Cockcroft-Gault formula (−2.9 mL/min in favor of everolimus, *P* = 0.46) [50]. Results of the extension phase in 81 patients demonstrated that everolimus maintained better renal function at 35 months (difference in eGFR between everolimus and CNI arms: Cockcroft-Gault: −10.5 mL/min, *P* = 0.096 and Nankivell formula: −10.5 mL/min, *P* = 0.015) [91].

In the fourth early-conversion prospective, randomized, high quality study (Table 3(b)) [87], 719 *de novo* liver transplant recipients were given a 30-day run-in period with tacrolimus-based immunosuppression (± mycophenolate) and then randomized to the following groups: everolimus (trough concentration, C0: 3–8 ng/mL) plus reduced-exposure tacrolimus (TAC-RD, C0: 3–5 ng/mL; *n* = 245); everolimus (C0: 6–10 ng/mL) with tacrolimus being withdrawn (TAC-WD, *n* = 231) at 4 months or standard-exposure tacrolimus (TAC-SD, C0: 6–10 ng/mL, *n* = 243). Enrolment in the TAC-WD arm was stopped prematurely due to a higher incidence of biopsy-proven acute rejection (BPAR) around the time of tacrolimus elimination. However, renal function at 1 year post-randomization improved significantly with everolimus plus TAC-RD, with an adjusted mean difference in eGFR change of +8.5 mL/min/1.73 m^2^ (97.5% confidence interval (CI) 3.74, 13.27 mL/min/1.73 m^2^) versus that observed in patients in the TAC-SD group (*P* < 0.001). The recent publication of final results from this trial demonstrates that significantly better renal function with TAC-RD was maintained at 24 months (mean difference in eGFR change: 6.66 mL/min/1.73 m^2^ (97.5% CI: 1.9, 11.42; *P* = 0.0018)) [90].

Of four late-conversion studies involving everolimus (Table 3(b)), two prospective, single-arm studies and one retrospective study demonstrated a benefit [94, 95, 98]. The other, a prospective, randomized, multicenter, medium quality study that involved administering everolimus with CNI reduction or discontinuation to 72 liver transplant recipients experiencing CNI-related renal impairment, failed to show a significant improvement in renal function from baseline or when compared to renal function in 73 CNI controls [96, 100]. A number of confounding factors were noted by the authors that may have potentially contributed to the negative result, including low CNI exposure at baseline due to previous efforts by the clinical team to improve renal function, and the fact that the extent of CNI dose reductions in the control group was higher than expected for a maintenance population.

Proteinuria was reported in four everolimus (two high quality and two low quality) studies identified in the literature search (Table 3(d)), occurring with incidences of 2.9% [87], 5.4% [98], 9.9% [50] and 29% [99]. One of these was a prospective randomized study that compared liver transplant recipients who received CNIs with/without corticosteroids (*n* = 102) to those who were switched early to everolimus with/without corticosteroids (*n* = 101). The incidence of proteinuria at 11 months post-randomization was higher in the everolimus group (9.9%) compared to the CNI group (2%), although six out of ten cases were mild with the remaining cases being moderate [50]. In another large prospective randomized high quality trial, in which patients were converted to everolimus early, proteinuria was observed in the everolimus plus reduced tacrolimus dose group during a 12-month followup period, but the maximum mean values for urinary protein to creatinine ratio were below 0.3 g/g, and preexisting cases of proteinuria did not worsen [87].

### 3.3. Safety of mTOR Inhibitors 

#### 3.3.1. Hepatic Artery Thrombosis (HAT)

*Sirolimus.* Two multicenter randomized studies in *de novo* liver transplant recipients suggested that the use of sirolimus in combination with cyclosporine or tacrolimus was associated with an increase in HAT [38, 44]. In subsequent studies (a mixture of quality and trial designs) that reviewed the use of sirolimus in liver transplant recipients, increased rates of HAT have not been observed (Table 4(a)) [15, 45, 48, 53, 55, 57, 61]. In fact, two of these studies recorded significantly lower incidences of HAT among patients receiving sirolimus compared to controls [53, 57]. In these studies, sirolimus was given at 2 mg per day without a loading dose, and sirolimus levels were targeted at 5–10 ng/mL, with long-term levels of 4–8 ng/mL [57], and in Molinari et al.'s study, sirolimus was maintained at 10–15 ng/mL during the first 3–6 months and 5–10 ng/mL thereafter [53].

*Everolimus.* An increased incidence of HAT was not observed in *de novo* [52] or prospective, randomized, high quality early-conversion [50, 87, 89] trials that included a control group (Table 4(a)). In one of the early-conversion studies, in which liver transplant recipients received everolimus at 2 mg with a target trough level of less than 12 ng/mL, the rate of hepatic artery stenosis/thrombosis was significantly lower when compared to a control group receiving cyclosporine (1.9 versus 15.4%, *P* = 0.04) [89].

#### 3.3.2. Portal Vein Thrombosis

*Sirolimus.* From four retrospective studies (two high quality studies and one medium and one low quality study) in which cases of portal vein thrombosis were monitored, three studies demonstrated no difference in the incidence among liver transplant recipients receiving sirolimus compared to those receiving CNI treatment (Table 4(a)) [15, 53, 55]. In the fourth study (high quality), there was a significantly lower incidence of portal vein thrombosis in 42 liver transplant recipients converted early from CNI treatment to sirolimus compared to 40 recipients in the CNI maintenance group (0% versus 8%, *P* = 0.02) [48]. In that study, sirolimus dosing targets for the first 3 months after conversion were 8–10 ng/dL and 6–8 ng/dL for months 3–6, and 5–6 ng/dL after month 12 [48].

#### 3.3.3. Wound Complications

*Sirolimus.* In seven studies, wound complications (described as either wound complications, wound dehiscence, wound infection, healing complications, or slow wound healing) were reported in 2.2–15% of liver transplant recipients receiving sirolimus (Table 4(b)) [15, 48, 53, 55, 61, 69, 78]. Six of these studies included a control group, of which five (a mixture of quality and trial designs) demonstrated no significant difference in wound complications between sirolimus and control groups [15, 53, 55, 61, 69, 78] and one small study demonstrated a significantly lower incidence of poor wound healing versus the CNI group (*P* = 0.017) [48]. Two of the studies reported that the incidence of incisional hernia was also similar in liver transplant recipients receiving sirolimus or CNIs [48, 53]. Only one study reported a higher incidence of wound infection in 111 liver transplant recipients receiving sirolimus-based immunosuppression compared to 52 CNI-based controls; however, in that open-label, prospective randomized study, the sirolimus dose was fixed at 5 mg per day [44].

*Everolimus.* Three prospective, randomized, high quality studies reported wound complications in liver transplant recipients receiving everolimus. In two of these studies, the incidence of wound complications and incisional hernia was similar in liver transplant recipients receiving everolimus compared to those receiving CNIs [50, 87]. The third study reported a higher rate of incisional hernia in 52 liver transplant recipients receiving everolimus compared to 26 recipients on cyclosporine (46.1% versus 26.9%), although the difference was not significant [89]. In these three studies, the initial everolimus dose did not exceed 3 mg per day and was then adjusted to achieve a target trough level of 5–12 ng/mL [50] or 6–10 ng/mL [87, 89].

#### 3.3.4. Edema

*Sirolimus.* Edema has been reported in 5–33% of liver transplant recipients receiving sirolimus (Table 4(b)) [45, 67, 71, 72, 75, 78, 80]. In two of these studies (both late-conversion studies, one high quality prospective randomized study and the other low quality retrospective study) edema occurred at significantly higher rates in liver transplant recipients receiving sirolimus compared to those receiving a CNI [45, 72].

*Everolimus.* Edema has been reported in 7–17.6% of liver transplant recipients receiving everolimus (Table 4(c)) [87, 89, 98, 99]. In one high quality prospective randomized trial, there was a higher relative risk (1.63, 95% CI: 1.03, 2.56) of peripheral edema in liver transplant recipients receiving everolimus plus reduced-dose tacrolimus (*n* = 245) compared to those receiving tacrolimus at a standard dose (*n* = 243) [87].

#### 3.3.5. Infections

*Sirolimus.* In 252 liver transplant recipients who received sirolimus as *de novo* immunosuppression, there were similar rates of infection compared to 291 recipients who received a CNI control [53]. In this retrospective, low quality study, there were also no differences in rates of herpes virus pneumonia, cytomegalovirus (CMV), or opportunistic infections within 6 months of liver transplantation [53]. In one early-conversion study and one *de novo* study that compared the incidence of CMV between liver transplant recipients receiving sirolimus and those receiving alternative immunosuppression, there were no significant differences [48, 57], and interestingly in one of these studies, there was a trend to less CMV viremia in the sirolimus group versus the control group (13.3% versus 20.2%, *P* = 0.07), Table 4(a)) [57]. Similarly, in a small prospective, late-conversion (medium quality) study, there was no significant difference in the rates of infections compared with the control group (Table 4(b)) [73].

*Everolimus.* In a prospective randomized, medium quality, early conversion study that used an initial dose of 3 mg twice daily, a similar incidence of infection occurred in recipients receiving everolimus compared to recipients receiving CNI treatment (31.9% versus 21.9%, *P* = nonsignificant). Most infections were stomatitis, nasopharyngitis, herpes simplex, bronchitis, or urinary tract infections. However, the incidence of infections that were suspected as being related to the study drug was higher in the everolimus patients (15.3%) compared to those receiving CNI treatment (1.4%) [96]. In contrast, prospective randomized studies (one low quality *de novo* and two high quality early conversion) administering everolimus at lower doses and without a higher initial dose reported a comparable incidence of infection for patients receiving CNIs and those receiving everolimus (Table 4(c)) [50, 52, 87]. Importantly, the incidence of CMV in similar studies was reported as not significantly different from control groups (Table 4(b)) [52, 87, 89].

#### 3.3.6. Oral Ulcers

*Sirolimus.* The development of oral ulcers has been reported in 9.5–42.0% of patients receiving sirolimus (Table 4(b)) [45, 48, 67, 73, 75, 77, 78, 80, 83] across a mixture of quality and trial designs. Mouth ulcers have been reported as occurring at a significantly higher rate in patients receiving sirolimus as compared to those receiving MMF [73] or CNI [45, 48], although it should be noted that two of these studies used high loading doses (≥10 mg) [45, 73].

*Everolimus.* Our literature search retrieved only one comparative study that reported oral ulcers—a prospective randomized, medium quality study that used a high initial dose (3 mg b.i.d) (Table 4(c)). In this study, mouth ulcers were reported to occur more frequently in 72 recipients receiving everolimus compared to 73 recipients receiving CNIs (26.4% versus 0.0%, *P* < 0.01) [96].

#### 3.3.7. Hematological Adverse Events

*Sirolimus.* Hematological adverse events have been reported in noncomparative studies at varying rates (Table 4(b)). Thrombocytopenia has been reported in up to 23% of patients [49, 69, 78, 81, 83], anemia in up to 23.8% [49, 67, 69, 83], and leukopenia in up to 25.7% [49, 69, 83]. In comparative studies, there were significant increases in the rates of anemia in one retrospective high quality and one prospective, randomized low quality study [45, 48] and leucopenia [45] was reported in patients receiving sirolimus versus CNIs, although two other studies (one retrospective high quality and one prospective, randomized medium quality) found no significant difference in anemia [72] and thrombocytopenia [78].

*Everolimus.* Hematological disturbances have been reported with the use of everolimus in six prospective studies (Table 4(c)), four of which included a control arm [50, 52, 87, 96]. In three of these variable-quality studies, the incidences of anemia, leukopenia, and thrombocytopenia in the everolimus groups were higher than in the control groups, although differences did not reach statistical significance [52, 87, 96]. Higher incidences of anemia, leukopenia, and thrombocytopenia were also reported in a high quality study of 101 recipients receiving everolimus compared to 102 recipients receiving CNI treatment, with leukopenia showing statistical significance [50]. A higher relative risk of leukopenia (2.38, 95% CI: 1.24, 4.55) was also reported in liver transplant recipients receiving everolimus plus reduced-dose tacrolimus compared to those receiving tacrolimus at a standard dose (randomized, high quality study) [87].

#### 3.3.8. Dermatological Adverse Events

*Sirolimus.* Skin rashes were reported in 7–69% of patients (Table 4(b)) [45, 48, 67, 75, 78, 80, 83]. In a retrospective conversion study (high quality) that compared the incidence of skin rashes between liver transplant recipients receiving sirolimus versus those receiving CNI treatment there were no significant differences [48].

*Everolimus.* Skin rashes have been reported with incidences of 6.9–19% (Table 4(c)) [95, 96, 98, 99]. In one of these studies, a prospective randomized medium quality study using an initial everolimus dose of 3 mg (b.i.d), skin rashes and eczema were reported more frequently in liver transplant recipients receiving everolimus compared to recipients receiving CNIs (skin rash: 6.9% versus 0.0%, *P* = 0.028; and eczema: 6.9% versus 0.0%, *P* = 0.028) [96].

### 3.4. The Effects of mTOR Inhibitors on Metabolic Syndrome

#### 3.4.1. New-Onset Diabetes Mellitus (NODM)

*Sirolimus.* Our literature search identified four studies that included observations on the effects of sirolimus on diabetes mellitus (Table 5). In the retrospective maintenance (low quality) study, a higher incidence of NODM was observed in a group of 65 patients receiving sirolimus for a 3-month period at some point following transplantation compared to those receiving CNI treatment (*n* = 49; 32% versus 10%, *P* = 0.005) [66]. This contrasted with a high quality case-control review of prospectively collected data among HCC patients in which the incidence of NODM was significantly higher in the tacrolimus + MMF group (*n* = 106, 12.26%) versus the sirolimus group (*n* = 121, 0%, *P* < 0.001) [55]. In two other retrospective studies (one late-conversion, medium quality and one *de novo*, low quality study), the incidence of diabetes reported in liver transplant recipients receiving sirolimus was similar to controls who had never received sirolimus [61] or did not change after conversion to sirolimus [75].

*Everolimus.* Two large, prospective, high quality early-conversion studies that compared liver transplant recipients who received everolimus or CNI treatment found no significant differences in the incidence of diabetes mellitus [50] or NODM (Table 5) [87].

Two studies investigated diabetes after late conversion to everolimus. One of these studies was a single-center, retrospective, medium quality study in which 62 liver transplant recipients were converted to everolimus-based immunosuppression. Of 18 patients with diabetes mellitus, seven showed an improvement in their condition; no information on the status of the remaining 11 patients was given [97]. In the other late-conversion study (retrospective and low quality), the incidence of diabetes did not change significantly after converting from CNI treatment to everolimus [99].

#### 3.4.2. Hypertension

Our search identified six studies (all conversion) that measured hypertension in liver transplant recipients receiving mTOR inhibitors (Table 5) [50, 75, 77, 78, 87, 99]. Of these six, one single-center prospective, single-arm, medium quality study demonstrated improvements in both diastolic and systolic blood pressure in 12 liver transplant recipients who had hypertension and had converted from CNI treatment to sirolimus. After conversion to sirolimus, it was possible to reduce the number of antihypertensive medications from two to one in four patients, and to stop antihypertensive medications in three patients [77]. In the other five studies, either the incidence of hypertension did not change after conversion from a CNI to sirolimus (both retrospective, late conversion studies) [75, 99] or there were no significant differences in hypertension or antihypertensive therapy requirements in recipients receiving an mTOR inhibitor versus those receiving CNIs [50, 78, 87] (prospective, randomized studies; two high quality everolimus and one medium quality sirolimus) (Table 5).

#### 3.4.3. Dyslipidemia

*Sirolimus.* Ten studies demonstrated that sirolimus use is associated with increases in serum lipid levels (Table 5) [45, 48, 73–76, 78, 80, 81, 84]. For example, in a small prospective study (medium quality) hypertriglyceridemia was significantly higher in a group who converted late to sirolimus from CNIs (*n* = 12) compared to those who converted to MMF (*n* = 13) [73]. In a large, prospective, randomized (low quality) study in which liver transplant recipients were abruptly converted to high-dose sirolimus from CNI treatment, the rates of hyperlipidemia and hypercholesterolemia were significantly higher than those in the recipients receiving CNI treatment (hyperlipidemia: 41% versus 10% and hypercholesterolemia: 28% versus 4%; both *P* < 0.001) [45]. In contrast, in a retrospective, early-conversion (high quality) study that used a lower sirolimus dose, hypertriglyceridemia (>200 mg/dL) was comparable between liver transplant recipients receiving sirolimus and those receiving CNI treatment [48].

*Everolimus.* Several studies of various designs report elevated lipid levels in liver transplant recipients (between 7 and 43%) receiving everolimus (Table 5) [52, 87, 89, 92–95, 95, 96, 98–100, 103, 104]. For example, in a prospective, randomized study in which *de novo* everolimus was given at either 1, 2, or 4 mg per day [52], there were dose-dependent increases from baseline in mean total cholesterol and triglyceride levels in the everolimus groups, with maximum levels reached by 6 months, although changes were not significantly different between treatment groups in this low quality study [52]. In a prospective, randomized, high quality conversion study, total cholesterol levels in the everolimus group were significantly higher than those in the control group at all observation timepoints up to 12 months of followup (1, 2, 3, 6 and 12 months) [89]. There were also increases in mean blood triglycerides levels in both groups, but this was not significant at any timepoint during followup [89]. Similarly, in a prospective, randomized, medium quality study that converted liver transplant recipients late from a CNI treatment to everolimus, hypercholesterolemia was significantly higher in patients receiving everolimus compared to recipients who remained on a CNI (13.9% versus 2.7%, *P* = 0.017) [96].

#### 3.4.4. Weight Gain

We identified three studies in the literature that examined the effect of sirolimus on body weight in liver transplant recipients, two of which suggested a benefit on weight gain after transplantation [61, 101] (Table 5). For example, a retrospective, low quality study showed that 170 liver transplant recipients who received *de novo* sirolimus had a significantly lower incidence of obesity (defined as body mass index (BMI) >28, *P* < 0.05) compared to a group of 180 historic controls who had received immunosuppression free of sirolimus [61]. The sirolimus group also had a lower BMI (25.5 versus 26.1 kg/m^2^), although this did not reach statistical significance [61]. Another retrospective, low quality study identified 210 liver transplant recipients who received *de novo* sirolimus, 567 recipients who received sirolimus as an addition to existing immunosuppression, and a control group of 777 recipients who had never received sirolimus [101]. Median weight was significantly lower at 2 and 5 years in the sirolimus group compared to the control group (2 years: 75.3 kg versus 84.1 kg, *P* = 0.05; 5 years: 79.5 kg versus 88.6 kg, *P* = 0.04), although recipients receiving sirolimus were slightly older (54 versus 50 years, *P* = 0.0001). In a third, prospective, medium quality study in which 13 liver transplant recipients were converted late to sirolimus from CNI treatment, there was no significant difference in the incidence of weight gain in recipients receiving sirolimus compared to 14 recipients receiving CNI treatment at 12 months after conversion (7.7% versus 0%) [78].

### 3.5. The Effects of mTOR Inhibitors in Patients with Concomitant HCC

Our literature search retrieved six studies examining the effect of sirolimus on HCC (Table 6); no studies were found for everolimus. Of these, four studies compared long-term survival in liver transplant recipients with HCC who received sirolimus compared to those who received sirolimus-free immunosuppression [15, 55, 59, 65]. For example, in one retrospective (low quality) study [15], overall survival at 1 and 5 years posttransplant for patients receiving sirolimus was higher than that for patients receiving standard CNIs (1 year: 95.5% versus 83%; 5 year: 80% versus 62%). The mortality risk ratio (sirolimus: CNIs) was 0.672 (*P* = 0.087). Importantly, HCC-recurrence-free survival at 1 and 5 years posttransplant in patients treated with sirolimus was higher (1 year: 93% versus 75%; 5 year: 78.8% versus 54%). The mortality HCC recurrence risk ratio (sirolimus: CNIs) was 0.622 and significant (*P* = 0.03) [15]. There were significantly higher recurrence-free survival rates (*P* = 0.001) and patient survival rates (80% versus 59%; *P* = 0.001 at 5 years posttransplantation) in the sirolimus group compared to the tacrolimus group in a retrospective, single-centre, high quality study in which a prospectively maintained database of liver transplants for HCC was reviewed. Patients who exceeded the Milan criteria were excluded and those who met the inclusion criteria received tacrolimus and MMF (*n* = 106) or sirolimus (*n* = 121) [55]. In a low quality study that analyzed data from the SRTR, consisting of 2,491 adults who had received liver transplantation for HCC, there was significantly improved survival in patients who had received sirolimus-based immunosuppression compared to those on sirolimus-free therapy at 5 years posttransplant (*P* ≤ 0.05) [65].

A retrospective, medium quality study of the recurrence rate and survival of patients after liver transplant for HCC exceeding the Milan criteria compared 27 patients treated with sirolimus-based immunosuppression with 46 patients who received tacrolimus-based treatment (Table 6) [59]. The overall survival of the sirolimus group was significantly higher than the tacrolimus group at 1 and 2 years posttransplantation (*P* = 0.011, log rank test, Table 6). Although no significant difference was observed between the two groups with respect to recurrence rate after 6 months and disease-free survival at 1 and 2 years, posttransplant recurrence in the sirolimus group occurred significantly later than in the tacrolimus group (258.7 ± 93.6 versus 144.0 ± 118.2 days; *P* = 0.036) [59].

### 3.6. The Effects of mTOR Inhibitors on HCV Recurrence and Fibrosis Progression in HCV Patients Receiving Liver Transplants (HCV-LT)

We identified nine studies that examined HCV progression in HCV-LT recipients receiving an mTOR inhibitor (Table 7). Only one study (*de novo*, prospective, randomized, medium quality) showed a benefit on HCV viral replication [58]. Four studies (all *de novo*; a mixture of retrospective and prospective; three medium quality, one low quality) demonstrated a benefit on fibrosis [56–58, 60], while four (a mixture of quality and trial designs) showed comparable results between liver transplant recipients receiving mTOR inhibitors and those receiving an alternative immunosuppressant [79, 88, 89, 102].

Studies that demonstrated a benefit of mTOR inhibitors on fibrosis included a retrospective, *de novo* study (medium quality) of 141 patients who underwent a first liver transplant for HCV cirrhosis [56]. There were no significant differences in patient survival, incidence of HCV recurrence, and inflammatory activity/fibrosis scores at the time of initial recurrence in patients treated with sirolimus compared to those who were sirolimus-free. However, in the sirolimus group, after a median followup of 16.6 months in patients with recurrence, the mean fibrosis scores on serial biopsies were significantly lower and the change in mean activity showed a decrease that approached significance [56]. In a large retrospective (medium quality) review of a prospectively maintained database, although patient/graft survival and HCV replication were similar between HCV recipients exposed to *de novo* sirolimus compared to HCV liver recipients who had never received sirolimus, protocol biopsies showed that the sirolimus group had reached a significantly reduced mean fibrosis stage at both 1 and 2 years posttransplant compared to the HCV control group. There was also a significantly lower incidence of advanced fibrosis (stage ≥2) in the sirolimus group at 1 and 2 years posttransplant. Stratifying the sirolimus group for the duration of sirolimus therapy showed that increases in the duration resulted in progressively less fibrosis [57]. In a small prospective, medium quality study that followed HCV-positive liver transplant recipients who received either sirolimus or CNIs [58], patients in the sirolimus group demonstrated a significant decrease from baseline in viral load at 12 months of followup, and viral load was significantly lower at 12 months compared to that in the CNI group [58]. In addition, although patient survival rates were comparable in both groups, a Kaplan-Meier survival analysis showed a trend towards better survival in the sirolimus group. In contrast to these survival results, a recent study analyzed patient survival in 26,414 liver transplant recipients from the SRTR database, of whom 12,589 were HCV-positive [105]. After adjusting for factors associated with mortality (including hepatocellular carcinoma, previous malignancy, hemodialysis, diabetes, gender mismatch, and other factors known to be related to patient outcomes), an increased risk of 3-year mortality was shown to be associated with the use of sirolimus at discharge in HCV-positive recipients, whereas non-HCV recipients had no increased risk related to sirolimus use [105].

### 3.7. The Effects of mTOR Inhibitors on Neurological Symptoms

Our literature search retrieved six studies, four for sirolimus [64, 70, 75, 80] and two for everolimus [89, 92], examining neurological symptoms in liver transplant recipients receiving immunosuppression with mTOR inhibitors (Table 8). In three of these studies, patients were switched to mTOR inhibitors because of neurological adverse events associated with the use of CNIs. All six studies showed a benefit with respect to neurological symptoms. For example, in a small, low quality retrospective study, seven liver transplant recipients were converted within 60 days of transplant to sirolimus because of CNI-related neurotoxicity [70]. Indications for conversion were peripheral neuropathy, seizure, metabolic encephalopathy, and central pontine myelinolysis. All patients showed improvement or resolution of their neurological symptoms [70]. In a single-center retrospective, low quality study, 16 liver transplant recipients were converted to sirolimus owing to neurotoxicity associated with CNI use [80]. The indications for conversion were dysarthria (*n* = 6), ataxia (*n* = 1), paralysis (*n* = 1), motor weakness (*n* = 1), tremor (*n* = 4), and psychosis (*n* = 5). Complete resolution was observed in 14 of these patients, while partial recovery was observed in the patient with ataxia [80]. In six liver transplant recipients who suffered from CNI-related neurotoxicity (severe headache, *n* = 1; invalidating tremor, *n* = 1; chronic partial epilepsy, *n* = 1; generalized tonic-clonic crisis in patients who had severe encephalopathy and pontic myelinolysis before transplant, *n* = 3) all patients had improvement of neurological symptoms after conversion [75].

The results of two small conversion studies (one high quality and one medium quality) suggested a benefit of everolimus with respect to neurological symptoms [89, 92]. In the first study, the incidence of minor neurological complications, defined as tremor, headache, or peripheral neuropathy, was lower in liver transplant recipients converted to everolimus monotherapy (5.8% of 52 recipients) compared to recipients receiving cyclosporine (19.2% of 26 recipients), although this difference was not significant [89]. In the second study, two cases of cerebrovascular stroke and one of Guillain-Barré syndrome associated with CNI use, everolimus use resulted in resolution or stabilization of these conditions [92].

### 3.8. Effect of mTOR Inhibitors on *De Novo* Tumors

A study that analyzed 33,249 renal transplant recipients from the Organ Procurement and Transplantation Network (OPTN) database has demonstrated that maintenance immunosuppression with sirolimus and everolimus is associated with a significantly reduced risk of developing any posttransplant *de novo* malignancy [106]. Our literature search did not find any equivalent study in liver transplant recipients. However, in a recent large prospective randomized conversion study (high quality), there was a small, nonsignificant difference in neoplasms (benign, malignant and unspecified, including cysts and polyps), with a neoplasm incidence of 4.0% reported in the everolimus group compared to 7.8% in the CNI group [50]. Three small studies were identified from our literature search that examined the effects of switching liver transplant recipients with *de novo* tumors to sirolimus [62, 80] or to everolimus [92] (Table 8). All three studies (one medium quality and two low quality) suggested a benefit of mTOR inhibitors with respect to survival in these patients.

### 3.9. Ongoing Clinical Trials on the Use of mTOR Inhibitors in Liver Transplantation

Eight ongoing clinical trials on the use of mTOR inhibitors in liver transplantation are currently listed on ClinicalTrials.gov. Study aims include evaluating the effect of everolimus on efficacy, safety, and renal function (NCT01150097, NCT01423708, NCT01625377, NCT01707849, and NCT01551212); the effect of sirolimus on HCC recurrence-free survival in patients following liver transplantation for HCC (NCT00355862, NCT01374750, and NCT00554125); and the impact of everolimus on HCV-related fibrosis (NCT01150097, NCT01707849, and NCT01551212).

## 4. Discussion

The results of our review indicate that the use of mTOR inhibitors, either as conversion or *de novo* immunosuppression, results in generally low rates of patient death, graft failure, and acute rejection that are comparable to those observed with CNIs and MMF. The majority of studies that we retrieved involved conversion to sirolimus or everolimus and most of these demonstrated a benefit on renal function, with some evidence that benefits are sustained for several years of followup. Converting to mTOR inhibitors within 3 months of transplantation was associated with better renal function than converting later, presumably since irreversible glomerular damage caused by chronic CNI use was limited [48, 49, 68, 80]. Renal function data for mTOR inhibitor use as *de novo* immunosuppression were more mixed with only one study out of four demonstrating a modest benefit of sirolimus on renal function [15], while two demonstrated no difference from control groups [52, 55].

Given that the use of CNIs, particularly tacrolimus, has been associated with an increase in the incidence of malignancies posttransplantation [27, 28, 107], the use of mTOR inhibitors with concomitant reduction or elimination of CNIs would be expected to reduce this risk. Antineoplastic effects have also been proposed for mTOR inhibitors [108–110] and everolimus marketed as Afinitor (Novartis Pharma AG; Basel, Switzerland) is already approved for a number of cancer indications and may have further relevance in the setting of posttransplant malignancy and HCC recurrence. Comparative studies identified in our review suggest that sirolimus-based immunosuppression is associated with higher rates of survival after liver transplantation for HCC compared to an alternative immunosuppressant [15, 55, 65]; mTOR inhibitors thus show promise as the preferred immunosuppressive agent in patients transplanted for end-stage liver disease with HCC and further randomized trials are currently underway that should increase the evidence base for the use of mTOR inhibitors in this patient subset (NCT00355862, NCT01374750, and NCT00554125). With regard to *de novo* malignancies, our search did not retrieve any studies that recorded the risk of developing posttransplant *de novo* malignancies. However, evidence from three studies suggests that survival is not adversely affected by sirolimus or everolimus [62, 80, 92]. It would be useful to further investigate the role of mTOR inhibition in this regard; although, to our knowledge, no such studies are currently being conducted.

Our review found some evidence of a benefit for mTOR inhibitor use in terms of reducing neurological symptoms. However, larger randomized studies are needed to determine whether mTOR inhibitors have a benefit with regard to neurotoxicity. To this end, one large randomized phase III trial (NCT01150097) is currently evaluating long-term safety and efficacy, including rates of neurotoxicity, of two concentration-controlled everolimus regimens in *de novo* liver transplant recipients.

Some experimental data support the concept that mTOR inhibitors confer a benefit in HCV-infected liver grafts [19, 111] However, a recent study advised caution against the use of sirolimus in HCV-LT patients [105]. This study was based on an analysis of the SRTR database and therefore there was a lack of information on HCV disease severity, length of sirolimus treatment and the dose/levels used, and HCV-specific outcomes, such as viral load, histological data, antiviral therapy, and whether the cause of death was attributable to recurrent hepatitis C. Our review of the literature found two studies that suggested a benefit for sirolimus on fibrosis progression in allograft biopsies from HCV-LT patients [56, 57]. In contrast, apart from one small study of short duration [58], there was no evidence that mTOR inhibitors reduce HCV replication. However, the ability to inhibit fibrosis in HCV-LT patients is a higher priority and critical for long-term survival. A number of randomized controlled studies that are currently ongoing (NCT01150097, NCT01707849, and NCT01551212) will help to define the role of mTOR inhibitors in HCV-related allograft fibrosis, particularly for everolimus, which has not been previously investigated in this regard.

Our review also suggests that mTOR inhibitors may reduce the risk of metabolic syndrome, as a positive effect was seen on all elements of the syndrome, with the exception of hyperlipidemia. The potential implications of this are substantial in light of the negative impact of metabolic syndrome on long-term survival. These results are encouraging but will need confirmation in larger randomized trials. One trial (NCT01150097) currently underway is investigating the effects of everolimus with CNI elimination/reduction on renal function in *de novo* liver transplant recipients and will also provide more information on the role of mTOR inhibitors on posttransplant diabetes and hypertension. There was also evidence that infections were no more common in liver transplant recipients receiving mTOR inhibitors compared to controls. Importantly, the incidence of CMV, which is the most frequent cause of viral infection in liver transplantation [112], was similar in liver transplant recipients receiving mTOR inhibitors compared to those receiving an alternative immunosuppressant, and in one study there was a trend to a lower incidence for sirolimus [57].

Concern about the use of mTOR inhibitors in liver transplantation stemmed from two studies that reported an apparent link between HAT and regimens including sirolimus plus cyclosporine or tacrolimus [38, 44]. Importantly we found no increase in the incidence of HAT in any of the other studies that we retrieved for everolimus and sirolimus. The occurrence of HAT, wound healing and severe proteinuria appears to be associated with higher doses or trough levels of mTOR inhibitors [44, 86]. Many current programs use lower doses of mTOR inhibitors than were used in older studies. In addition, mTOR-inhibitor treatment is generally initiated without a loading dose, whereas the use of a loading dose (up to 15 mg in the case of sirolimus) was a common practice historically. Due to an increase in more-serious adverse events with higher doses of sirolimus, without any additional benefits on efficacy, McKenna and Trotter [46] have proposed that a sirolimus dose of 2 mg daily without the use of a loading dose and serum levels of 4–10 ng/mL should be used. Adverse events have also been shown to increase with increasing doses of everolimus, especially at ≥4 mg/day [52], suggesting that they could therefore be minimized by reducing the dose used.

Both mTOR inhibitors were associated with elevated lipid levels, mouth ulcers, edema, skin rashes, and hematological disturbances (thrombocytopenia, leukopenia, and anemia) [50, 67]. These adverse effects present a challenge to the clinician as they can preclude mTOR inhibitor use due to the negative impact they have on long-term patient outcomes and quality of life. However, with appropriate monitoring and clinical intervention, many of the adverse effects seen with mTOR inhibitors are manageable; for example, the dyslipidemia that occurs with mTOR inhibitors can be managed with lipid-lowering drugs, and mouth ulcers can be effectively treated with topical kenalog-in-orabase.

## 5. Conclusion

The use of mTOR inhibitors in liver transplantation is associated with benefits on renal function and efficacy comparable to CNIs. By using appropriate protocols (i.e., controlling the dose to minimize toxicity and, when mTOR inhibitors are used as conversion therapy, converting within 3 months of transplant to enable prevention of CNI-related renal dysfunction) serious adverse events can be limited and renal function optimized. Though mouth ulcers, skin rashes, hypercholesterolemia, and hematological disturbances are common with mTOR inhibitor use, they are generally manageable. Our results also suggest that mTOR inhibitors have additional benefits in the oncology setting and potential benefits on HCV-related allograft fibrosis, metabolic syndrome, and neurotoxicity, which could distinguish this class of immunosuppressants and have important long-term implications for liver transplant patients.

## Figures and Tables

**Table:** (a)

	Participants	Study design^a^	Duration of study	Sirolimus dosing	Strength of study design based on defined criteria^b^
*De novo* dosing					
Chinnakotla et al. *Liver Transpl.* 2009; 15: 1834–42 [55]	*N* = 227 recipients (i) *n* = 121 (SRL) (ii) *n* = 106 (TAC)	R/P	5 years	2 mg orally, once daily. Levels maintained at 5–8 ng/mL for the first 3 months and at 5 ng/mL thereafter	High
Asthana et al. *Can J Gastroenterol. *2011; 25: 28–34 [56]	*N* = 141 recipients (i) *n* = 88 (*de novo* SRL) (ii) *n* = 53 (CNI)	R	12 months	Maintained at 8–12 ng/mL	Medium
McKenna et al. *Am J Transpl. *2011; 11: 2379–87 [57]	*N* = 455 recipients (HCV-positive) (i) *n* = 173 (SRL) (ii) *n* = 282 (SRL-free)	R/P	2 years	2 mg daily without a loading dose beginning on the first postoperative day	Medium
Wagner et al. *Int Immunopharmacol.* 2010; 10: 990–3 [58]	*N* = 67 recipients (HCV-positive) (i) *n* = 39 (SRL) (ii) *n* = 28 (CNI)	P, C	12 months	Trough levels were maintained between 3 and 8 ng/mL	Medium
Zhou et al. *Transplant Proc*. 2008; 40: 3548–553 [59]	*N* = 73 recipients (HCC exceeding Milan criteria) (i) *n* = 27 (SRL) (ii) *n* = 46 (TAC)	R	2 years	Given 1 month after transplant: initial dose of 3 mg/m^2^ adjusted over time to achieve steady-state trough levels of *≈*5–8 ng/mL	Medium
Asthana et al. Presented at AASLD 2011 (Abstract 184) [60]	*N* = 100 recipients (recurrent HCV) (i) *n* = 40 (SRL) (ii) *n* = 35 (TAC) (iii) *n* = 24 (cyclosporine) (iv) *n* = 36 lost to followup	P, C	Median of 77.6 months	Not stated	Low
Campsen et al. *J Transplant.* 2011; 2011: 913094 [54]	*N* = 672 recipients (i) *n* = 328 (CNI + MPS at time of discharge) (ii) *n* = 135 (CNI + MPS at time of discharge; SRL added within the first 6 months and continued through the first year) (iii) *n* = 15 (CNI + MPS at time of discharge; SRL was added within the first 6 months and discontinued before the first year) (iv) *n* = 156 (SRL as primary immunosuppression) (v) *n* = 38 (SRL as primary immunosuppression and discontinued before the first year)	R	1 year	Not stated	Low
Dunkelberg et al. *Liver Transpl. *2003; 9: 463–8 [61]	*N* = 350 recipients (i) *n* = 170 (SRL) (ii) *n* = 180 (historic controls)	R	12 months	6 mg on day 0, and 2 mg/day thereafter no target level was specified	Low
Jiménez-Romero et al. *Hepatogastroenterology.* 2011; 58: 115–21 [62]	*N* = 16 recipients who developed *de novo* tumors All switched from CNI/MMF to SRL monotherapy	P, S	Mean of 15.7 months	Loading dose: 4 mg, followed by 2 mg/day until 8–12 days, thereafter dose adjusted to achieve target blood level of 5–10 ng/mL	Low
Kneteman et al. *Liver Transpl.* 2004; 10: 1301–11 [63]	*N* = 40 recipients (HCC) All given SRL	P, S	4 years	Adjusted to achieve target levels of 12–20 ng/mL	Low
Maramattom and Wijdicks*Neurology.* 2004; 63: 1958–9 [64]	*N* = 202 recipients All received SRL	R	18 months	Loading dose: 6 mg, thereafter: 1–10 mg/day with target blood level of 8–15 ng/mL	Low
Molinari et al. *Transpl Intl.* 2010; 23: 155–68 [53]	*N* = 543 recipients (i) *n* = 252 (SRL) (ii) *n* = 291 (CNI)	R	5 years	Oral dose adjusted to keep the blood levels in the range of 10–15 ng/mL during the first 3–6 months and then in the range of 5–10 ng/mL afterwards	Low
Toso et al. *Hepatology.* 2010; 51: 1237–43 [65]	*N* = 2491 recipients (HCC) (i) *n* = 109 (SRL) (ii) *n* = 2382 (SRL-free)	R	5 years	Not stated	Low
Wiesner et al. *Am J Transplant.* 2002; 2 (s3): 464 (Abstract 1294) [44]	*N* = 163 recipients (i) *n* = 111 (SRL + CsA) (ii) *n* = 52 (concentration-controlled TAC (trough levels 5–15 ng/mL) + corticosteroids)	P, Ra	6 months	Fixed-dose of 5 mg/day	Low
Zimmerman et al. *Liver Transpl.* 2008; 14: 633–8 [15]	*N* = 97 recipients (cirrhosis + concomitant HCC) (i) *n* = 45 (SRL) (ii) *n* = 52 (standard regimen including CNIs, MMF, and corticosteroids)	R	5 years	Bolus dose of 6 mg on day 0 and given on 2 mg/day thereafter	Low

Combination of *de novo* and maintenance dosing					
Kazimi et al. *Transplantation.* 2010; 90 (2S): 697 (Abstract 1950) [66]	*N* = 114 recipients (i) *n* = 65 (SRL) (ii) *n* = 49 (CNI)	R	1111 ± 800 days	Not stated	Low

**Table:** (b)

	Participants	Study design^a^	Duration of study	Sirolimus dosing	Strength of study design based on criteria^b^
Early conversion (≤3 months after transplantation)					
Rogers et al. *Clin Transplant.* 2009; 23: 887–96 [48]	*N* = 82 recipients (i) *n* = 42 (SRL) (ii) *n* = 40 (CNI)	R	12 months	Target SRL levels for the first three months after conversion were 8–10 ng/dL, 6–8 ng/dL for months 3–6, and 5–6 ng/dL after month 12	High
Harper et al. *Transplantation*. 2011; 91: 128–32 [67]	*N* = 148 recipients All converted to SRL	R	Median of 1006 days	Conversion from CNI to SRL performed with an overlap. SRL given at 2 mg/day and CNI withdrawn when SRL reached 5–8 ng/mL	Medium
McKenna et al. *ILTS.* 2011 (Abstract O-17) [68]	*N* = 1078 recipients (i) *n* = 202 (SRL) (ii) *n* = 876 (SRL-free)	R	10 years	Not stated	Medium
Schleicher et al. *Transplant Proc*. 2010; 42: 2572–5 [49]	*N* = 57 recipients (i) *n* = 11 (early conversion, impaired perioperative renal function; SRL = 7, EVL = 4) (ii) *n* = 7 (early conversion, normal perioperative renal function; SRL = 6, EVL = 1) (iii) *n* = 23 (late conversion, impaired perioperative renal function, SRL = 15, EVL = 8) (iv) *n* = 16 (late conversion, normal perioperative renal function; SRL = 12, EVL = 4)	R	12 months	Initial dose of 10 mg/day. Doses were adjusted successively to maintain trough levels of 5–10 ng/mL	Medium
Sanchez et al. *Transplant Proc*. 2005; 37: 4416–23 [69]	*N* = 64 recipients (i) *n* = 29 (*denovo* SRL) (ii) *n* = 35 (conversion SRL)	P, C	2 years	In recipients with HCC or autoimmune disorders, SRL doses used were typically either a 5 mg or 3 mg loading dose followed by 2 mg each day After conversion, SRL levels were maintained at 10–15 ng/mL when used within 3 months of transplantation and at 5–10 ng/mL after 3 months after transplantation	Medium
Forgacs et al. *Transplant Proc*. 2005; 37: 1912–4 [70]	*N* = 7 recipients All converted to SRL	R	Up to 425 days	Not stated	Low

Late conversion (>3 months after transplantation)					
Campbell et al*. Clin Transplant*. 2007; 21: 377–84 [71]	*N* = 179 recipients (i) *n* = 79 (SRL conversion) (ii) *n* = 100 (CNI controls)	R	Median of 359 days	2 mg daily, dose adjusted until target levels of 5–8 ng/mL achieved	High
DuBay et al. *Liver Transpl*. 2008; 14: 651–9 [72]	*N* = 114 recipients (with renal insufficiency) (i) *n* = 57 (SRL) (ii) *n* = 47 (low-dose CNIs)	R	12 months	Recipients on CNI monotherapy were started on SRL 1 mg/day and the CNI dose was halved. At 1 week, the CNI was stopped, and the SRL dose was adjusted on the basis of the serum levels. For recipients on combination therapy, the CNI was stopped, and SRL was started on the same day at 2 mg/day while the antimetabolite or steroid doses were maintained at their current levels. In both groups the SRL dose was adjusted to maintain trough levels of 5–15 *μ*g/day	High
Herlenius et al. *Transplant Proc*. 2010; 42: 4441–8 [73]	*N* = 25 recipients (with chronic kidney disease) (i) *n* = 12 (SRL) (ii) *n* = 13 (MMF)	P, Ra	12 months	A single bolus dose of 10 mg SRL followed by three consecutive daily doses of 8 mg. Target trough concentration of 10 ng/mL	Medium
Lam et al. *Dig Dis Sci*. 2004; 49: 1029–35 [74]	*N* = 28 recipients (with renal insufficiency after transplantation) All converted to SRL	P, S	Mean of 328 ± 57 days	SRL initiated at 2 mg/day. Doses adjusted to achieve target level of 4–10 ng/mL	Medium
Morard et al. *Liver Transpl*. 2007; 13: 658–64 [75]	*N* = 48 recipients (i) *n* = 16 (SRL) (ii) *n* = 19 (SRL + MMF) (iii) *n* = 7 (SRL + prednisone) (iv) *n* = 2 (SRL + CNI) (v) *n* = 4 (SRL + MMF + prednisone)	R	Median of 22.6 ± 11 months	Loading dose: 6 mg (day 1) followed by 2 mg/day (day 2–7). SRL dose adjusted to maintain trough levels of 5–10 ng/mL	Medium
Shenoy et al*.Transplantation*. 2007; 83: 1389–92 [76]	*N* = 40 recipients (with renal dysfunction) (i) *n* = 20 (SRL) (ii) *n* = 20 (CNI)	P, Ra	12 months	5 mg loading dose, followed by 3 mg SRL once daily. Levels maintained 6–10 ng/mL	Medium
Uhlmann et al. *Exp Clin Transplant.* 2012; 10: 30–8 [77]	*N* = 25 recipients All converted to SRL	P, S	75.6 months	SRL started at 1 mg/day, dose adjusted to maintain trough levels at 69 ng/mL	Medium
Watson et al. *Liver Transpl*. 2007; 13: 1694–702 [78]	*N* = 27 recipients (i) *n* = 13 (SRL) (ii) *n* = 14 (CNI)	P, Ra	12 months	CNI was discontinued the evening before conversion, and recipients were started on 2 mg/day SRL on the following day. Target range of 5–15 ng/mL	Medium
Stein et al. Presented at the American Transplant Congress 2011 (Abstract 817) [79]	*N* = 40 recipients (received transplant for HCV) (i) *n* = 18 (SRL conversion) (ii) *n* = 22 (TAC)	R	5 years	Not stated	Low
Vivarelli et al.*Transplant Proc*. 2010; 42: 2579–84 [80]	*N* = 78 recipients (i) *n* = 38 (SRL) (ii) *n* = 40 (SRL + CNI)	R	510 ± 366 days	5 mg/m^2^ for 1st day, then 2 mg/daily, adjusted to trough blood level <10 ng/mL	Low
Di Benedetto et al*.Transplant Proc*. 2009; 41: 1297–9 [81]	*N* = 31 recipients All converted to SRL	R	Mean of 27.5 months (range: 2–71.2 months)	SRL at a loading dose of 0.1 mg/kg on day 1 of the switch, then 0.05 mg/kg for the next few days. Dose adjusted to maintain trough levels of 8–10 ng/mL	Low
Abdelmalek et al*. Am J Transplant*. 2012; 12: 694–705 [45]	*N* = 607 recipients (i) *n* = 393 (SRL conversion) (ii) *n* = 214 (CNI continuation)	P, Ra	Up to 6 years	Loading dose of SRL: 10–15 mg. First dose given ≥4 h after the last CNI dose; second doses given 12 h later. On study days 2–6, SRL doses of 3–5 mg/day were given. Thereafter, SRL doses maintained to achieve blood levels of 6–16 ng/mL (chromatographic) and subsequently to 8–16 ng/mL (chromatographic) or 10–20 ng/mL (immunoassay)	Low
Bäckman et al.* Clin Transplant*. 2006; 20: 336–9 [82]	*N* = 15 recipients All converted to SRL	P, S	6 months	Loading dose of 15 mg SRL on days 1 and 2, then 8 mg/day and adjusted to achieve trough levels of 13–22 and 10–22 ng/mL	Low
Fairbanks et al*. Liver Transpl*. 2003; 9: 1079–85 [83]	*N* = 21 recipients (developed renal dysfunction while on CNI therapy) All converted to SRL	P, S	Mean of 66.8 ± 38.9 weeks	Initially 1-2 mg/day and increased weekly by 1 mg to achieve therapeutic levels (9–12 ng/mL)	Low
Nair et al*. Liver Transpl*. 2003; 9: 126–9 [84]	*N* = 16 recipients All converted to SRL	R	6 months	Loading dose of 5 mg on day 1, followed by 2 mg/day. A trough level of 5–10 ng/mL was maintained	Low
Neff et al*. Transplant Proc*. 2003; 35: 3029–31 [85]	*N* = 14 recipients All converted to SRL	R	90 days	Mean starting dose of SRL (10 mg/day) adjusted to maintain trough levels of 8–12 ng/mL during first month and subsequently 3–5 ng/mL for recipients on maintenance combination therapy with SRL	Low
Wadei et al*. Transplantation*. 2012; 93: 1006–12 [86]	*N* = 102 recipients All converted to SRL	R	Median of 3.1 years	CNI dose reduced by 50% until target SRL level of 8–12 ng/mL achieved	Low

Search terms were ‘‘sirolimus liver transplantation” OR ‘‘sirolimus liver transplant.”

CNI: calcineurin inhibitor; HCC: hepatocellular carcinoma; HCV: hepatitis C virus; ILTS: 2011 Joint International Congress of the International Liver Transplantation Society; MMF: mycophenolate mofetil; SRL: sirolimus; TAC: tacrolimus.

^a^Study design: C: cohort; P: prospective; R: retrospective; Ra: randomized; S: single-arm.

^b^See Section 2 for description of how criteria are defined.

**Table:** (c)

	Participants	Study design^a^	Study duration	Everolimus dosing	Strength of study design based on criteria^b^
De Simone et al. *Am J Transplant*. 2012; 12: 3008–20 [87]	*N* = 719 recipients (i) *n* = 245 (EVR + TAC-RD) (ii) *n* = 231 (EVR + TAC-WD) (iii) *n* = 243 (TAC-SD)	P, Ra	12 months	For EVR + TAC-WD, EVR initiated at a dose of 1.0 mg b.i.d. within 24 h of randomization with the dose adjusted from day 5 onward, to maintain *C*0 3–8 ng/mL until month 4 after transplantation, after which the target range increased to 6–10 ng/mL In the EVR + TAC-RD arm, EVR initiated and monitored as for EVR + TAC-WD, but the initial target range of 3–8 ng/mL maintained throughout the study	High
Grazi et al. Presented at ILTS; 2011 (Abstract P-256) [88]	*N* = 64 recipients (HCV-related cirrhosis) (i) *n* = 28 (EVR + Bmab + steroids) (ii) *n* = 36 (TAC + Bmab + steroids)	P, Ra	1 year	Not stated	Low
Levy et al. Liver Transpl. 2006; 12: 1640–8 [52]	*N* = 119 recipients (i) *n* = 28 (EVR 1.0 mg) + CNI (ii) *n* = 30 (EVR 2.0 mg) + CNI (iii) *n* = 31 (EVR 4.0 mg) + CNI (iv) *n* = 30 (placebo) + CNI	P, Ra	36 months	1, 2 or 4 mg/day	Low

Search terms were ‘‘everolimus liver transplantation” OR ‘‘everolimus liver transplant.”

Maintenance therapy refers to immunotherapy for the lifetime of the graft. Conversion therapy is where liver transplant recipients were withdrawn from CNIs and switched to everolimus.

Bmab: basiliximab; CNI: calcineurin inhibitor; EVR: everolimus; HCV: hepatitis C virus; SRL: sirolimus; TAC: tacrolimus.

^a^Study design: P: prospective; R: retrospective; Ra: randomized.

^b^See Section 2 for description of how criteria are defined.

**Table:** (d)

	Participants	Study design^a^	Study duration	Everolimus dosing	Strength of study design based on criteria^b^
Early conversion (≤3 months after transplantation)					
De Simone et al*. Am J Transplant*. 2012; 12: 3008–20 [87]	*N* = 719 recipients (i) *n* = 245 (EVR + TAC-RD) (ii) *n* = 231 (EVR + TAC-WD) (iii) *n* = 243 (TAC-SD)	P, Ra	12 months	For EVR + TAC-WD, EVR initiated at a dose of 1.0 mg b.i.d. within 24 h of randomization with the dose adjusted from day 5 onward, to maintain *C*0 3–8 ng/mL until month 4 after transplantation, after which the target range increased to 6–10 ng/mL. In the EVR + TAC-RD arm, EVR initiated and monitored as for EVR + TAC-WD, but the initial target range of 3–8 ng/mL maintained throughout the study	High
Fischer et al*. Am J Transplant*. 2012; 12: 1855–65 [50]	*N* = 203 recipients on CNI with/out corticosteroids (i) *n* = 101 (EVR) (ii) *n* = 102 (CNI continuation)	P, Ra	12 months	EVR started at 1.5 mg b.i.d. and adjusted to achieve a target trough level of 5–12 ng/mL (8–12 ng/mL in patients on treatment with CsA), when CNI was tapered by 70% of the initial CNI dose	High
Masetti et al*. Am J Transplant*. 2010; 10: 2252–62 [89]	*N* = 78 recipients (i) *n* = 52 (EVR) (ii) *n* = 26 (CsA)	P, Ra	12 months	Initial dose: 2.0 mg/day, trough level of 6–10 ng/mL. When CsA discontinued, trough level: 8–12 ng/mL until end of month 6 and 6–10 ng/mL thereafter	High
Saliba et al. AASLD 2012 [90]	*N* = 719 recipients from De Simone et al. *Am J Transplant.* 2012 [40] (i) *n* = 245 (EVR + TAC-RD) (ii) *n* = 231 (EVR + TAC-WD) (iii) *n* = 243 (TAC-SD)	P, Ra	24 months	Same as De Simone et al. *Am J Transplant.* 2012 [40]	High
Schlitt et al. AASLD 2012 [91]	*N* = 81 recipients from Fischer et al. *Am J Transplant*. 2012 (i) *n* = 41 (EVR with/out corticosteroids) (ii) *n* = 40 (CNI with/out corticosteroids)	P, Ra	35 months	Same as Fischer et al. *Am J Transplant*. 2012 [43]	High

Late conversion (>3 months after transplantation)					
Bilbao et al*. Transplant Proc*. 2009; 41: 2172–6 [92]	*N* = 25 recipients All converted to EVR	R	Mean of 10 ± 9 months	In refractory rejection: initial dose 0.5 mg/12 h. Trough levels 5 ng/mL. For CNI-related adverse events, EVR started at 0.5 mg once or twice a day. For malignancy, EVR introduced at 0.5 mg/day, adjusting trough levels to <3 ng/mL	Medium
Casanovas et al*. Transplant Proc*. 2011; 43: 2216–9 [93]	*N* = 35 recipients All converted to EVR	P, S	Mean of 134 months	Initial dose 0.25 mg/12 h for the first 4 days. Target trough 3–5 ng/mL	Medium
Castroagudín et al. *Liver Transpl*. 2009; 15: 1792–7 [94]	*N* = 21 recipients (chronic renal dysfunction) All converted to EVR	P, S	Median of 19.8 months	0.75 mg b.i.d., with target trough levels of 3–8 ng/mL	Medium
De Simone et al*. Transpl Int*. 2009; 22: 279–86 [95]	*N* = 40 recipients	P, S	12 months	EVR 1.5 mg/day. Trough level of 3–8 ng/mL	Medium
De Simone et al*. Liver Transpl*. 2009; 15: 1262–9 [96]	*N* = 145 recipients (i) *n* = 72 (EVR therapy with CNI reduction or discontinuation) (ii) *n* = 73 (CNI continuation)	P, Ra	12 months	Initial: 3 mg/day ×2 on day 1. After week 2: EVR trough level maintained at 3–8 ng/mL during concomitant CNI administration and 6–12 ng/mL if CNI eliminated	Medium
Bilbao et al. Presented at ILTS; 2011 (Abstract P-68) [97]	*N* = 62 recipients All received EVR	R	Median of 12 months	EVR trough level at ~3 ng/mL	Low
Saliba et al*. Liver Transpl*. 2011; 17: 905–13 [98]	*N* = 240 maintenance recipients All received EVR	R	12 months	Introduced at mean 2.4 mg/day. The mean trough level = 7.3 ng/mL at month 1 and 8.1 ng/mL at month 12 across total population, with higher values in monotherapy cohort (8.8 ng/mL at month 12)	Low
Vallin et al*. Clin Transplant*. 2011; 25: 660–9 [99]	*N* = 94 recipients All received EVR	R	Mean of 12 ± 7 months	Initial dose 0.75–1.5 mg b.i.d. Trough adjusted to 3–8 ng/mL	Low

*P* values are included where available.

Search terms were ‘‘everolimus liver transplantation” OR ‘‘everolimus liver transplant.”

AASLD: Annual Meetings of the American Association for the Study of Liver Diseases; b.i.d.: twice daily; CNI: calcineurin inhibitor; CsA: cyclosporin A; EVR: everolimus; ILTS: 2011 Joint International Congress of the International Liver Transplantation Society; TAC: tacrolimus; TAC-RD: reduced-dose tacrolimus; TAC-SD: standard-dose tacrolimus; TAC-WD: tacrolimus withdrawn.

^a^Study design: P: prospective; R: retrospective; Ra: randomized; S: single-arm.

^b^See Section 2 for description of how criteria are defined.

**Table:** (a)

	Patient survival rate (%)			Graft rejection rate (%)		Acute rejection rate (%)	BPAR (%)	Acute cellular rejection rate		
	6 months	1 year	Crude mortality rate/1000 person-months	Crude graft failure rate/1000 person-months	1 year			(%)	ACR/1000 person-months	Steroid-resistant rejection/1000 person-months
*De novo* dosing										
Sanchez et al*. Transplant Proc*. 2005; 37: 4416–23 [69]								17.2		
Molinari et al*. Transpl Intl.* 2010; 23: 155–68 [53]	95 versus 94 (SRL versus CNI)	92 versus 91 (SRL versus CNI)			0 versus 0 (SRL versus CNI)		32.5 versus 44.1 (SRL versus CNI, *P* = 0.03)	46.7 versus 58.9 (SRL versus CNI, *P* = 0.003)		
Campsen et al*. J Transplant*. 2011; 2011: 913094 [54] *See footnote for groups			2.39 (group 1), 1.96 and 3.88 (SRL conversion groups 2 and 3), 1.82 and 2.13 (SRL *de novo* groups 4 and 5). All NS	2.68 (group 1), 2.15 and 3.88 (SRL conversion groups 2 and 3), 1.82 and 2.13 (SRL *de novo* groups 4 and 5). All NS					12.5 (group 1), 13.4, 12.9 (SRL conversion groups 2, 3, *P* = NS versus control), 6.5, 5.2 (SRL *de novo *groups 4, 5; *P* ≤ 0.0001, *P* = 0.0007 versus controls)	3.48 (group 1), 4.29, 3.88 (SRL conversion groups 2, 3, *P* = 0.03, *P* = NS versus controls), 1.58, 1.54 (SRL *de novo *groups 4, 5; *P* < 0.001, *P* = 0.04 versus controls)

Late conversion (>3 months after transplantation)										
DuBay et al. *Liver Transpl*. 2008; 14: 651–9 [72]						5 versus 4 (SRL versus control)				
Shenoy et al. *Transplantation*. 2007; 83: 1389–92 [76]						5 versus 5 (SRL versus control)				
Watson et al. *Liver Transpl*. 2007; 13: 1694–702 [78]						7.7 versus 0 (SRL versus CNI)				
Abdelmalek et al. *Am J Transplant*. 2012; 12: 694–705 [45] (Low)		93.4 versus 94.4 (SRL versus CNI)			0 versus 0 (SRL versus CNI)	6.4 versus 1.9 (SRL versus CNI)	11.7 versus 6.1 (SRL versus CNI, *P* = 0.02)			
Bäckman et al. *Clin Transplant*. 2006; 20: 336–9 [82]						6.7				
Di Benedetto et al. *Transplant Proc*. 2009; 41: 1297–9 [81]							12.9			
Fairbanks et al. *Liver Transpl*. 2003; 9: 1079–85 [83]						4.8				
Nair et al*. Liver Transpl*. 2003; 9: 126–9 [84]								0		

Conversion times compared										
Rogers et al*.Clin Transplant.* 2009; 23: 887–96 [48]							35, 38, 43 (early, late conversion SRL and CNI)			

Variable conversion times										
Harper et al*. Transplantation*. 2011; 15; 91: 128–32 [67]							3.4			
Sanchez et al. *Transplant Proc*. 2005; 37: 4416–23 [69]								2.8		

*P*  values are included where available.

ACR: acute cellular rejection; BPAR: biopsy-proven acute rejection; CNI: calcineurin inhibitor; NS: nonsignificant; SRL: sirolimus.

*Key to groups:

(1) CNI + MPS at time of discharge.

(2) CNI + MPS at time of discharge; SRL added within the first 6 months and continued through the first year.

(3) CNI + MPS at time of discharge; SRL was added within the first 6 months and discontinued before the first year.

(4) SRL as primary immunosuppression.

(5) SRL as primary immunosuppression and discontinued before the first year.

**Table:** (b)

	Patient survival rate (%)			Graft rejection rate (%)			Acute rejection rate (%)	BPAR (%)		
	6 months	1 year	Other time points	6 months	1 year	3 years		6 months	1 year	Other time points
*De novo*/maintenance dosing										
De Simone et al.* Am J Transplant*. 2012; 12: 3008–20 [87]		96.3 versus 97.5 (EVR + TAC-RD versus TAC-SD, *P* = NS)			2.4 versus 1.2 (EVR + TAC-RD versus TAC-SD, *P* = NS)				4.1 versus 10.7 (EVR + TAC-RD versus TAC-SD, *P* = 0.005)	
Grazi et al. Presented at ILTS; 2011 (Abstract P-256) [88]										
Levy et al. *Liver Transpl*. 2006; 12: 1640–8 [52] (Low) + CNI		82.1, 96.7, 87.1 for EVR 1, 2 and 4 mg/day^∗†^			0, 13.3 and 3.2 for EVR 1, 2 and 4 mg/day^∗†^	39.3, 30.0 and 29.0 for EVR 1, 2 and 4 mg/day^∗†^			32.1, 26.7, and 25.8 for EVR 1, 2 and, 4 mg/day^∗†^	39.3, 30.0, and 29.0 for EVR 1, 2, and 4 mg/day^∗†^ at 3 years

Early conversion (≤3 months after transplantation)										
Fischer et al. *Am J Transplant*. 2012; 12: 1855–1865 [50]		95.8 versus 95.9 (EVR versus CNI control at 11 months, *P* = NS)			2.1 versus 2.0 (EVR versus CNI control at 11 months)					17.7 versus 15.3 (EVR versus CNI control at 11 months)
Schlitt et al. AASLD 2012 [91]			95.7 versus 90.0 (EVR versus CNI control at 35 months, *P* = NS)							24.4 versus 15.8 (EVR versus CNI control at 35 months, *P* = NS)
Masetti et al. *Am J Transplant*. 2010; 10: 2252–62 [89]	92.3 versus 92.3 (EVR versus CsA, *P* = NS)	90.4 versus 88.5 (EVR versus CsA, *P* = NS)								5.7 at 40–87 days after transplant versus 7.7 at days 41–240 after transplant CsA (*P* = NS)
De Simone et al. *Am J Transplant*. 2012; 12: 3008–3020 [87]		96.5 versus 96.3 versus 97.5 (TAC elim versus EVR + TAC-RD versus TAC-SD)			2.2 versus 2.4 versus 1.2 (TAC elim versus EVR + TAC-RD versus TAC-SD)				19.9 versus 4.1 versus 10.7 (TAC elim versus EVR + TAC-RD versus TAC-SD)	
Saliba et al. AASLD 2012 [90]										6.1 versus 13.3; delta risk: −7.2% (95% CI: −13.5%, −0.9%; *P* = 0.010. EVR+TAC-RD versus TAC-SD at 24 months)

Late conversion (>3 months after transplantation)										
Casanovas et al. *Transplant Proc*. 2011; 43: 2216–9 [93]		94.3								
Castroagudín et al. *Liver Transpl*. 2009; 15: 1792–7 [94]							0			
De Simone et al. *Transpl Int*. 2009; 22: 279–86 [95]		100			0				15	
De Simone et al. *Liver Transpl*. 2009; 15: 1262–9 [96]	98.6 versus 100 (EVR versus CNI)	95.8 versus 95.9 (EVR versus CNI)		0 versus 0 (EVR versus CNI)				1.4 versus 1.4 (EVR versus CNI)	4.2 versus 4.1 (EVR versus CNI)	
Saliba et al. *Liver Transpl*. 2011; 17: 905–13 [98]									1.6	
Vallin et al. *Clin Transplant*. 2011; 25: 660–9 [99]									9	

*P* values are included where available;_ _*P: not significant for all efficacy-related events versus placebo; otherwise, where not stated.

^†^Timepoint refers to time after immunosuppression was initiated.

AASLD: The Liver Meeting 62nd Annual Meeting of the American Association for the Study of Liver Diseases; BPAR: biopsy-proven acute rejection; CsA: cyclosporin A; CNI: calcineurin inhibitor; EVR: everolimus; NS: nonsignificant; TAC elim; tacrolimus elimination; TAC-RD: reduced dose tacrolimus (*C*0: 3–5 ng/mL); TAC-SD: standard-dose tacrolimus (*C*0: 6–10 ng/mL).

**Table:** (a)

	mTOR inhibitor	Change in GFR (mL/min/1.73 m^2^) after conversion					Change in serum creatinine concentration (mg/dL) after conversion		Change in CrCl (mL/min) from baseline at postconversion time point	
		3 months	6 months	1 year	2 years	5 years	6 months	1 year	6 months	1 year
Levy et al. *Liver Transpl*. 2006; 12: 1640–8 [52]	EVR								EVR: 1, 2, and 4 mg/day versus placebo: −18.7, −36.5, and −33.2 versus −37	EVR: 1, 2, and 4 mg/day versus placebo: −20.2, −43.0, and −36.9 versus −36.9
Chinnakotla et al*. Liver Transpl*. 2009; 15: 1834–1842 [55]	SRL	−46.5 versus −41.0 (SRL versus TAC)		−39.5 versus −30.0 (SRL versus TAC)	−43.5 versus −31.5 (SRL versus TAC)	−38.5 versus −37.0 (SRL versus TAC)				
Campsen et al*. J Transplant*. 2011; 2011: 913094 [54]	SRL		−2.85 versus −9.66 (CNI versus SRL, *P* = 0.0465)	−6.29 versus −13.32 (CNI versus SRL, *P* = 0.0320)						
Zimmerman et al*. Liver Transpl*. 2008; 14: 633–8 [15]	SRL						−0.22 versus +0.61 (SRL versus CNIs, *P* = 0.0021)	−0.28 versus +0.35 (SRL versus CNIs, *P* < 0.0001)		

*P* values are included where available.

CNI: calcineurin inhibitor; CrCl: creatinine clearance; EVR: everolimus; GFR: glomerular filtration rate; mTOR: mammalian target of rapamycin; SRL: sirolimus; TAC: tacrolimus.

**Table:** (b)

	Change in GFR (mL/min/1.73 m^2^) after conversion					Change in serum creatinine concentration (µm/L) after conversion						Change in 24 h CrCl (mL/min) after conversion	
	3 mo	6 mo	1 year	2 years	3 years	1 mo	3 mo	6 mo	1 year	2 years	3 years	3 mo	1 year
Early conversion (≤3 months after transplantation)													
McKenna et al*. ILTS.* 2011 (Abstract O-17) [68]													
Rogers et al*. Clin Transplant.* 2009; 23: 887–96 [48]	+29.7 versus +18.5 (early versus late conversion, *P* < 0.05)	+28.7 versus +12.4 (early versus late conversion, *P* < 0.05)	+25.5 versus +14.7 (early versus late conversion, *P* < 0.05)										
Harper et al. *Transplantation*. 2011; 91: 128–32 [67]					+10.6 versus baseline (*P* < 0.05)								
Schleicher et al. *Transplant Proc*. 2010; 42: 2572–5 [49]	+15 versus +9 (early versus late conversion, *P* = 0.04)	+16 versus +10 (early versus late conversion, *P* = 0.05)	+22^†^ versus +12^†^ (early versus late conversion, *P* = 0.04)						−0.7^‡^ (early conversion versus baseline, *P* = 0.01) −0.6^‡^ (late conversion versus baseline, *P* = 0.05)				

Late conversion (>3 months after transplantation)													
Campbell et al. *Clin Transplant*. 2007; 21: 377–84 [71]							0^‡^ (SRL versus CNI)						
Di Benedetto et al*. Transplant Proc*. 2009; 41: 1297–9 [81]													
DuBay et al*. Liver Transpl*. 2008; 14: 651–9 [72]													
Herlenius et al. *Transplant Proc*. 2010; 42: 4441–8 [73]			+12 versus baseline (*P* = 0.03)										
Lam et al*. Dig Dis Sci*. 2004; 49: 1029–35 [74]						−0.32^‡^ versus baseline (*P* = 0.029)		+0.12^‡^, versus baseline (*P* = NS)					
Sanchez et al*. Transplant Proc*. 2005; 37: 4416–23 [69]	+8.2, *P* = 0.05 versus case control		+20.2, NS versus case control	+16.1, NS versus case control			−0.1		0	0			
Shenoy et al. *Transplantation*. 2007; 83: 1389-92 [76]													+12 versus −4, NS versus CNI
Uhlmann et al*. Exp Clin Transplant.* 2012; 10: 30–8 [77]			+11.9 versus baseline (*P* < 0.05)						−27 versus baseline (*P* < 0.05)				
Watson et al. *Liver Transpl*. 2007; 13: 1694–702 [78]	+7.7 versus baseline (*P* = 0.001)		+6.1 versus baseline (*P* = 0.024)										
Abdelmalek et al.* Am J Transplant*. 2012; 12: 694–705 [45]			−4.45 versus −3.07 (SRL versus CNI, *P* = 0.34)										
Bäckman et al*. Clin Transplant*. 2006; 20: 336–9 [82]		+6.4 (*P* = NS from baseline)											
Fairbanks et al*. Liver Transpl*. 2003; 9: 1079–85 [83]			+9* versus baseline (*P* = 0.01)										
Nair et al*. Liver Transpl*. 2003; 9: 126–9 [84]								−0.45^‡^ versus baseline (*P* = 0.001)					
Neff et al*. Transplant Proc*. 2003; 35: 3029–31 [85]												+11.3 versus baseline	
Vivarelli et al. *Transplant Proc*. 2010; 42: 2579–84 [80]	+18 versus baseline^†^	+19 versus baseline^†^			+19 versus baseline^†^								
Wadei et al*. Transplantation*. 2012; 93: 1006–12 [86]					+3.5^†^ versus baseline (*P* = 0.03)						−0.1 versus baseline (*P* = 0.25)		

*P* values are included where available.

*Median eGFR, mean of 67 weeks; ^†^mL/min; ^‡^mg/dL.

CNI: calcineurin inhibitor; CrCl: creatinine clearance; GFR: glomerular filtration rate; mo: month; NS: nonsignificant; SRL: sirolimus.

**Table:** (c)

	Change in GFR (mL/min/1.73 m^2^) after conversion			Change in serum creatinine concentration (µmol/L) after conversion			Change in CrCl (mL/min) from baseline at postconversion time point	
	6 months	1 year	Other time points	3 months	6 months	1 year	6 months	1 year
Early conversion (≤3 months after transplantation)								
Fischer et al*. Am J Transplant*. 2012; 12: 1855–65 [50]		11-month data (EVR versus CNI): CG-GFR: mean change from baseline: + 4.4 ± 27.1 versus 0.9 ± 29.2 LS mean difference ±SE: −2.923 ± 3.920 (*P*=0.457)^§^ MDRD-GFR: mean change from baseline: +2.0 ± 23.2 versus −2.8 ± 23.1LS mean difference ± SE: −7.778 ± 3.338 (*P*=0.021)^§^						
Schlitt et al. AASLD 2012 [91]			35 months: difference in eGFR between EVR and CNI: Cockcroft-Gault: −10.5 mL/min (*P* = 0.096) and Nankivell formula: −10.5 mL/min (*P* = 0.015)					
Masetti et al*. Am J Transplant*. 2010; 10: 2252–62 [89]	+6.1 versus −16.5 (EVR versus CsA, *P* < 0.001 comparison of absolute values)	+5.9 versus −14.8 (EVR versus CsA, *P* < 0.001 comparison of absolute values)						
De Simone et al*. Am J Transplant*. 2012; 12: 3008–20 [87]		EVR + TAC-RD (adjusted mean difference in eGFR change for EVR + TAC-RD versus TAC-SD: +8.50 ± 2.12; *P* < 0.001)						
Saliba et al. AASLD 2012 [90]			24 months: EVR + TAC-RD versus TAC-SD: mean difference in eGFR change: +6.66 (97.5% CI: +1.9, + 11.42; *P* = 0.0018)					

Late conversion (>3 months after transplantation)								
Castroagudín et al*. Liver Transpl.* 2009; 15: 1792–7 [94]	+3.99 (NS versus baseline)	+7.65 (*P* = 0.016 versus baseline)			−0.09 (NS versus baseline)	−0.22^‡^ (*P* < 0.05 versus baseline)	+5.18 (NS versus baseline)	+9.82 (*P* = 0.025 versus baseline)
De Simone et al*. Transpl Int*. 2009; 22: 279–86 [95]								+4.03
De Simone et al*. Liver Transpl.* 2009; 15: 1262–9 [96]							EVR: +1.0; controls: +2.3 (NS)	
Saliba et al. *Liver Transpl*. 2011; 17: 905–13 [98]	+6.6 chronic renal failure subpopulation versus baseline (*P* = 0.0002)	+4.2 overall versus baseline (*P* = 0.007) +8.6 chronic renal failure subpopulation versus baseline (*P* = 0.02)		−11 versus baseline (*P* = 0.04)	−9 versus baseline (*P* = NS)	−6 versus baseline (*P* = NS)		

*P* values are included where available.

*Median eGFR; ^†^mL/min; ^‡^mg/dL.

^§^Between-group difference (calculated as CNI group minus everolimus group) at month 11 after baseline; results based on ANCOVA model.

CG-GFR: GFR calculated with the Cockcroft-Gault formula; CNI: calcineurin inhibitor; CrCl: creatinine clearance; CsA: cyclosporin A; EVR: everolimus; GFR: glomerular filtration rate; LS: least square; MDRD: modification of diet in renal disease; MDRD-GFR: GFR calculated using the MDRD formula; NS: nonsignificant; SE: standard error; TAC-RD: reduced-dose tacrolimus (*C*0: 3–5 ng/mL); TAC-SD: standard-dose tacrolimus (*C*0: 6–10 ng/mL).

**Table:** (d)

	Proteinuria (after conversion unless otherwise stated), % recipients
Sirolimus studies	

Early conversion (≤3 months after transplantation)	
Harper et al*. Transplantation.* 2011; 91: 128–32 [67]	Proteinuria: preconversion: 45; 6 months: 72; 3 years: 63; 5 years: 58

Late conversion (>3 months after transplantation)	
DuBay et al*. Liver Transpl*. 2008; 14: 651–9 [72]	7 versus 4 (SRL versus CNI)
Herlenius et al*. Transplant Proc*. 2010; 42: 4441–8 [73]	8.3 versus 7.7 (SRL versus MMF)
Morard et al*. Liver Transpl*. 2007; 13: 658–64 [75]	Albuminuria: 36
Uhlmann et al*. ExpClin Transplant.* 2012; 10: 30–8 [77]	Proteinuria increased at 6 months after conversion (*P* < 0.05)
Wadei et al*. Transplantation.* 2012; 93: 1006–12 [86]	81

Everolimus studies	

Early conversion (≤3 months after transplantation)	
De Simone et al*. Am J Transplant*. 2012; 12: 3008–20 [87]	EVR + TAC-RD versus TAC-SD: 2.9 versus 0.4 (RR: 6.89, 95% CI 0.85, 55.54)
Fischer et al. *Am J Transplant*. 2012; 12: 1855–65 [50]	EVR versus CNI: 9.9 versus 2.0

Late conversion (>3 months after transplantation)	
Castroagudín et al*. Liver Transpl*. 2009; 15: 1792–7 [94]	38.1
Saliba et al*. Liver Transpl*. 2011; 17: 905–13 [98]	5.4
Vallin et al*. Clin Transplant*. 2011; 25: 660–9 [99]	13 versus 29 (pre- versus after conversion, *P* < 0.05)

*P* values are included where available.

CNI: calcineurin inhibitor; EVR: everolimus; MMF: mycophenolate mofetil; SRL: sirolimus; RR: relative risk; 95% CI: 95% confidence interval; TAC: tacrolimus; TAC-RD: reduced-dose tacrolimus (*C*0: 3–5 ng/mL); TAC-SD: standard-dose tacrolimus (*C*0: 6–10 ng/mL).

**Table:** (a)

	mTOR inhibitor	HAT (%)	Portal vein thrombosis (%)
*De novo* dosing			
Levy et al. *Liver Transpl*. 2006; 12: 1640–8 [52]	EVR	Placebo: 3.3, EVR 1, 2, and 4 mg/day: 0, 3.3, and 3.2, respectively	
Chinnakotla et al. *Liver Transpl*. 2009; 15: 1834–42 [55]	SRL	1.65 versus 0 (SRL versus TAC, *P* = NS)	1.65 versus 0.94 (SRL versus TAC, *P* = NS)
Molinari et al. *Transpl Intl.* 2010; 23: 155–68 [53]	SRL	1.2 versus 5.8 (SRL versus CNI, *P* = 0.004)	0.8 versus 1.8 (SRL versus CNI, *P* = NS)
Dunkelberg et al. *Liver Transpl*. 2003; 9: 463–8 [61]	SRL	Hepatic artery complications: 5.3 versus 8.3 (SRL versus controls, *P* = NS)	
Wiesner et al. *Am J Transplant*. 2002; 2 (s3): 464 (Abstract 1294) [44]	SRL	8.1 versus 3.8 and 9.0 versus 3.8 (SRL + CsA + CS versus TAC + CS, 2 and 6 months after transplant, *P* = NS for both timepoints)	
Zimmerman et al. *Liver Transpl*. 2008; 14: 633–8 [15]	SRL	2.2 versus 1.9 (SRL versus CNI)	0 versus 0

Early conversion (≤3 months after conversion)			
De Simone et al. *Am J Transplant*. 2012; 12: 3008–20 [87]	EVR	0.4 versus 0.4 compared to 1.9 in all patients during the prerandomization run-in phase (EVR + TAC-RD versus EVR + TAC-WD)	
Masetti et al. *Am J Transplant*. 2010; 10: 2252–62 [89]	EVR	Hepatic artery stenosis/thrombosis: 1.9 versus 15.4 (EVR versus CsA, *P* = 0.04)	
McKenna et al. *Am J Transplant*. 2011; 11: 2379–87 [57]	SRL	1.2 versus 5.6 (SRL versus SRL-free, *P* = 0.02)	
Rogers et al. *Clin Transplant.* 2009; 23: 887–96 [48]	SRL	7 versus 11 (SRL versus CNI, *P* = NS)	0 versus 8 (*P* = 0.02)
Schleicher et al. Transplant Proc. 2010; 42: 2572–5 [49]	SRL	0	

Late conversion (>3 months after conversion)			
Abdelmalek et al.* Am J Transplant*. 2012; 12: 694–705 [45]	SRL	0.25 versus 0 (SRL versus CNI, *P* = NS)	

*P* values are included where available.

CNI: calcineurin inhibitor; CsA: cyclosporine A; EVR: everolimus; HAT: hepatic artery thrombosis; NS: nonsignificant; SRL: sirolimus; TAC: tacrolimus; TAC-RD: reduced-dose tacrolimus (*C*0: 3–5 ng/mL); TAC-WD: tacrolimus withdrawn.

**Table:** (b)

	Edema (% recipients)	Wound complications (% recipients)	Ulcers (% recipients)	Bile duct complications (% recipients)	Infections (% recipients)	Dermatological effects (% recipients)	Hematological effects (% recipients)
*De novo *dosing							
Chinnakotla et al. *Liver Transpl*. 2009; 15: 1834–1842 [55]		Wound dehiscence: 3.31 versus 1.89 (SRL versus TAC, *P* = NS)					
McKenna et al. *Am J Transplant*. 2011; 11: 2379–87 [57]					CMV: 13.3 versus 20.2 (SRL versus controls, *P* = 0.07)		
Sanchez et al. *Transplant Proc.* 2005; 37 (10): 4416–23 [69]		Wound infection: 13.8 Wound dehiscence: 6.9 (both *de novo*)			Bacterial infection: 34.5 versus 22.9 CMV: 3.4 versus 14.3 (*de novo *versus conversion)		Leukopenia: 6.9 versus 25.7 (*de novo *versus conversion) Thrombocytopenia: 13.7 versus 8.6 (*de novo *versus conversion)
Molinari et al. *Transpl Intl* 2010; 23: 155–68 [53]		Wound complications: 15 versus 11.6 (SRL versus CNI, *P* = NS) Incisional hernias: 8.7 versus 7.2 (*P* = NS)		19.4 versus 18.5 (SRL versus CNI, *P* = NS)	Opportunistic infections: 18.5 versus 13 CMN: 2.6 versus 2.7 Herpes virus pneumonia: 0 versus 0.9 (SRL versus CNI, 6 months, *P* = NS for all)		
Dunkelberg et al. *Liver Transpl.* 2003; 9: 463–8 [61]		Wound complications: 12.4 versus 13.9 (SRL versus controls, *P* = NS)					
Zimmerman et al. *Liver Transpl.* 2008; 14: 633–8 [15]		Wound infection: 2.2 versus 3.8 (SRL versus CNI)					

Early conversion (≤3 months after transplantation)							
Rogers et al. *Clin Transplant*. 2009; 23: 887–96 [48]		Incisional hernia: 14 versus 15 (SRL versus CNI, *P* = NS) Poor wound healing: 6 versus 20 (*P* = 0.017)	Oral ulcers: 15 versus 3 (SRL versus CNI, *P* = 0.019)		CMV disease: 13 versus 6 (SRL versus CNI, *P* = NS)	Facial rash: 8 versus 5 (SRL versus CNI, *P* = NS)	Leukopenia: 20 versus 12 (SRL versus CNI, *P* = NS) Anemia: 44 versus 11 (SRL versus CNI, *P* < 0.001)
Harper et al. *Transplantation*. 2011; 91: 128–32 [67]	Peripheral edema: 21		Mouth ulcer: 15		Pneumonitis: 5	Rash: 7	Anemia: 11
Schleicher et al. *Transplant Proc*. 2010; 42: 2572–5 [49]		Incisional hernia: 7					Leukopenia: 12 Thrombocytopenia: 10.5 Anemia: 9

Late conversion (>3 months after transplantation)							
Campbell et al. *Clin Transplant*. 2007; 21: 377–84 [71]	Leg edema: 5						
DuBay et al. *Liver Transpl*. 2008; 14: 651–9 [72]	Lower extremity edema: 23 versus 11 (SRL versus CNI, *P* = 0.08)						Anemia: 16 versus 12 (SRL versus CNI, *P* = NS)
Watson et al. *Liver Transpl*. 2007; 13: 1694–702 [78]	Edema: 30.8 versus 7.1 (SRL versus CNI)	Slow wound healing: 7.7 versus 0 (SRL versus CNI)	Oral ulcers: 38			Rash: 69 versus 0 (SRL versus CNI)	Thrombocytopenia: 23 versus 14.3 (SRL versus CNI)
Herlenius et al. *Transplant Proc*. 2010; 42: 4441–8 [73]			Oral ulcers: 42 versus 0 (SRL versus MMF, *P* = 0.01)		Infectious episode: 33.3 versus 38.5 (SRL versus MMF, *P* = NS)		
Morard et al. *Liver Transpl*. 2007; 13: 658–64 [75]	Ankle edema: 14		Oral ulcer: 12		Infection: 2	Dermatitis: 14	
Shenoy et al. *Transplantation*. 2007; 83: 1389–92 [76]						Mouth sores: 25 versus 0 (SRL versus CNI) Pruritus: 5 versus 0 (SRL versus CNI)	
Uhlmann et al. *Exp Clin Transplant*. 2012; 10: 30–8 [77]			Oral ulcer: 16				
Fairbanks et al. *Liver Transpl*. 2003; 9: 1079–85 [83] (Low)			Oral ulcer: 9.5			Rash: 9.5 Acne: 9.5	Anemia: 23.8 Leukopenia: 9.5 Thrombocytopenia: 14.3
Di Benedetto et al. *Transplant Proc*. 2009; 41: 1297–9 [81]							Thrombocytopenia (leading to withdrawal): 12.9
Abdelmalek et al. *Am J Transplant*. 2012; 12: 694–705 [45]	Peripheral edema: 33 versus 14 (SRL versus CNI, *P* < 0.001)		Oral ulcer: 11 versus 1 (SRL versus CNI, *P* < 0.001)		Herpes simpex: 9 versus 1 (SRL versus CNI, *P* < 0.001) Hepatitis: 2 versus 0 (SRL versus CNI, *P* = 0.03)	29 versus 8 (SRL versus CNI, *P* < 0.001)	
Vivarelli et al.* Transplant Proc*. 2010; 42: 2579–84 [80]	Lower limb edema: 23.3		Oral ulcer: 5.8			Rash: 5.8	Anemia: 13.9

*P* values are included where available.

CMV: cytomegalovirus; CNI: calcineurin inhibitor; MMF: mycophenolate mofetil; NS: non-significant; SRL: sirolimus; TAC: tacrolimus.

**Table:** (c)

	Edema (% recipients)	Wound complications (% recipients)	Ulcers (% recipients)	Bile duct complications (% recipients)	Infections (% recipients)	Dermatological effects (% recipients)	Hematological effects (% recipients)
*De novo* dosing							
Levy et al. *Liver Transpl*. 2006; 12: 1640–8 [52]					CMV disease: 3.3, 3.6, 6.7, and 9.7 (placebo versus EVR 1, 2, and 4 mg/day, respectively, *P* = NS for all comparisons)		Thrombocytopenia: 10.0, 14.3, 20.0, and 19.4 Leukopenia: 0, 14.3, 6.7, and 6.5 (placebo, EVR 1, 2, and 4 mg/day, PS = NS for all comparisons)

Early conversion (≤3 months after transplantation)							
De Simone et al. *Am J Transplant*. 2012; 12: 3008–20 [87]	17.6 versus 10.8 (EVR + TAC-RD versus TAC-SD, RR 1.63, 95% CI 1.03, 2.56)	Wound complications: 11.0 versus 7.9 (EVR + TAC-RD versus TAC-SD, RR 1.40, 95% CI 0.80, 2.45) Incisional hernia: 2.9 versus 1.2 (RR 2.30, 95% CI, 0.60, 8.77)					Leukopenia: 11.8 versus 5.0 (EVR + TAC-RD versus TAC-SD, RR 2.38, 95% CI 1.24, 4.55) Thrombocytopenia: 5.3 versus 1.7 Anemia: 7.8 versus 8.3 (EVR + TAC-RD versus TAC-SD, RR 0.93, 95% CI 0.51, 1.71)
Fischer et al. *Am J Transplant*. 2012; 12: 1855–65 [50]		Wound complications: 2 versus 3.9 Incisional hernia: 11.9 versus 9.8 Wound dehiscence: 0 versus 1 Wound hemorrhage: 1 versus 0 (all EVR versus CNI)			Infections and infestations: 73.3 versus 59.8 (EVR versus CNI)		Anemia: 18.8 versus 10.8 Leukopenia: 20.8 versus 9.8* Thrombocytopenia: 7.9 versus 6.9 (all EVR versus CNI, *P* = NS for anemia and thrombocytopenia)
Masetti et al. *Am J Transplant*. 2010; 10: 2252–62 [89]	Inferior limb edema: 9.6 versus 0 (EVR versus CsA, *P* = NS)	Incisional hernia: 46.1 versus 26.9 (EVR versus CsA, *P* = NS)		Biliary complications (stenosis/leak): 21.1 versus 30.8 (EVR versus CsA, *P* = NS)	Infections: 46.1 versus 46.1 (EVR versus CsA, *P* = NS) CMV: 19.2 versus 23.1 (EVR versus CsA, *P* = NS)		

Late conversion (>3 months after transplantation)							
Bilbao et al. *Transplant Proc*. 2009; 41: 2172–6 [92]			Mucositis: 4		Sepsis in the context of graft-versus-host disease: 4		
Casanovas et al. *Transplant Proc*. 2011; 43: 2216–9 [93]							Anemia, leukopenia, and thrombocytopenia: 11.4
De Simone et al. *Transpl Int*. 2009; 22: 279–86 [95]			Oral ulcers/stomatitis: 22.5		Lower urinary tract infection: 5	Pruritis and acne: 7.5	
De Simone et al. *Liver Transpl*. 2009; 15: 1262–9 [96]			Mouth ulcers: 26.4 versus 0.0 (EVR versus SNI, *P* < 0.01)		Infections: 31.9 versus 21.9, of which 15.3 versus 1.4 were suspected to be drug-related (EVR versus CNI)	Rash/dry skin/eczema: 6.9 versus 0.0 (EVR versus CNI, *P* = 0.028)	Leukopenia: 12.5 versus 5.5 Thrombocytopenia: 5.6 versus 1.4 Anemia: 9.7 versus 4.1 (all EVR versus CNI)
Saliba et al. *Liver Transpl*. 2011; 17: 905–13 [98]	Edema: 16.3		Stomatitis/mouth ulcers: 14.2		Bacterial infection: 12.5	Rash: 18.8	Anemia: 12.9 Leukopenia: 9.2 Thrombocytopenia: 6.3
Vallin et al. *Clin Transplant*. 2011; 25: 660–9 [99]	Edema: 7		Mucositis: 15		Infection: 3	Dermatitis: 19	

CMV: cytomegalovirus; CNI: calcineurin inhibitor; CsA: cyclosporine A; EVR: everolimus; NS: nonsignificant; SRL: sirolimus; RR: relative risk; 95% CI: 95% confidence interval; TAC: tacrolimus; TAC-RD: reduced-dose tacrolimus (*C*0: 3–5 ng/mL); TAC-SD: standard-dose tacrolimus (*C*0: 6–10 ng/mL).

*P* values are included where available *Treatment group differences with an exploratory *P* value of *P* ≤ 0.05.

**Table 5:** Effect of mTOR inhibitors on components of metabolic syndrome.

	NODM (% recipients)	Hypertension (% recipients)	Lipid levels (% recipients)	Weight gain (% recipients)
Sirolimus				

Maintenance dosing				
Kazimi et al. *Transplantation.* 2010; 90 (2S): 697 (Abstract 1950) [66]	32 versus 10 (SRL versus CNI, *P* = 0.005)			

*De novo* dosing				
Chinnakotla et al. *Liver Transpl*. 2009; 15: 1834–42 [55]	12.26 versus 0 (TAC versus SRL, *P* < 0.001)			
Levy et al. *Liver Transpl*. 2006; 12: 1640–8 [52]			Hypercholesterolemia: 3.3, 7.1, 10.0, 9.7	
Dunkelberg et al. *Liver Transpl*. 2003; 9: 463–8 [61]	26 versus 30 (SRL versus SRL-free, *P* = NS)			Obesity (BMI > 28): 20 versus 31 (SRL versus SRL-free, *P* < 0.05)

Early conversion (≤3 months after conversion)				
Rogers et al. *Clin Transplant.* 2009; 23: 887–96 [48]			Hypertriglyceridemia (>200 mg/dL): 22 versus 15 (SRL versus CNI, *P* = NS) Hypertriglyceridemia (>500 mg/dL): 13 versus 0 (*P* = 0.003)	
Schleicher et al. *Transplant Proc. * 2010; 42: 2572–5 [49]			Hyperlipidemia: 42	

Late conversion (>3 months after conversion)				
Herlenius et al. *Transplant Proc*. 2010; 42: 4441–8 [73]			Hypertriglyceridemia: 58.3 versus 16.7 (SRL versus MMF, *P* = 0.002)	
Lam et al. *Dig Dis Sci*. 2004; 49: 1029–35 [74]			Increase in serum cholesterol: 30.5 (*P* = 0.003)	
Shenoy et al. *Transpl.* 2007; 83: 1389–92 [76]			Hyperlipidemia requiring treatment: 15 (SRL and CNI)	
Uhlmann et al. *Exp Clin Transplant.* 2012; 10: 30–8 [77]		In 12 hypertensive patients who converted to SRL: systolic BP decreased from 151.5 ± 20.2 mmHg to 132.1 ± 19.4 mmHg, and diastolic BP decreased from 89.7 ± 11.2 mmHg to 82.1 ± 9.1 mmHg		
Morard et al. *Liver Transpl*. 2007; 13: 658–64 [75]	Incidence of diabetes did not change after conversion (32 versus 30, before versus after conversion)	Incidence of hypertension did not change after conversion (70 versus 74, before versus after conversion)	Hypercholesterolemia: 49 (*P* = NS)	
Watson et al. *Liver Transpl*. 2007; 13: 1694–702 [78]		Hypertension treatment increased: 15.4 versus 21.4 (SRL versus CNI, *P* = NS) Hypertension treatment reduced: 23.1 versus 0 (SRL versus CNI, *P* = NS)	Statin treatment required: 35.7 versus 0 (SRL versus CNI)	Weight gain: 7.7 versus 0 (SRL versus CNI, *P* = NS)
Vivarelli. *Transplant Proc*. 2010; 42: 2579–84 [80]			Hyperlipidemia: 51.1	
Abdelmalek et al. *Am J Transplant*. 2012; 12: 694–705 [45]			Hyperlipidemia: 41 versus 10 (SRL versus CNI, *P* < 0.001) Hypercholesterolemia: 28 versus 4 (SRL versus CNI, *P* < 0.001)	
Di Benedetto et al. *Transplant. Proc*. 2009; 41: 1297–9 [81]			Hypertriglyceridemia: 35.4 Hypercholesterolemia: 25.8	
McKenna et al. *Hepatology.* 2009; 50; 590A: Abstract 602 [101]				Median weight 2 years: 75.3 versus 84.1 (SRL versus non-SRL, *P* = 0.05) 5 years: 79.5 versus 88.6 (SRL versus non-SRL, *P* = 0.04)

Everolimus				

Early conversion (≤3 months after conversion)				
De Simone et al. *Am J Transplant*. 2012; 12: 3008–20 [87]	Incidence of NODM in non-diabetics at randomization in EVR + TAC-RD: 32.0% (*n* = 48/150) versus TAC-SD: 28.6% (*n* = 40/140) (*P* = 0.609)	17.1 versus 15.8 (EVR + TAC-RD versus TAC-SD, RR 1.09, 95% CI 0.73, 1.62)	Total cholesterol: mean (SD) values at month 12 (EVR + TAC-RD versus TAC-SD): 209 (43) mg/dL versus 175 (44) mg/dL (*P* < 0.001) Triglycerides: 197 (136) mg/dL versus 141 (78) mg/dL (*P* < 0.001)	
Fischer et al. *Am J Transplant*. 2012; 12: 1855–65 [50]	Diabetes mellitus: 4.0 versus 7.8 (EVR versus CNI, *P* = NS)	Hypertension: 19.8 versus 13.7 (EVR versus CNI, *P* = NS)	Hypercholesterolemia: 22.8 versus 10.8* Hyperlipidemia: 11.9 versus 2.0* (EVR versus CNI)	
Masetti et al. *Am J Transplant*. 2010; 10: 2252–62 [89]				Significantly higher cholesterol levels in EVR versus CsA: 9.6 versus 7.7 required statin treatment (EVR versus CsA)

Late conversion (>3 months after conversion)				
Bilbao et al. Presented at ILTS; 2011 (Abstract P-68) [56]	7/18 recipients who had diabetes mellitus at conversion to EVR showed improvement			
Casanovas et al. *Transplant Proc*. 2011; 43: 2216–9 [93]			Dyslipidemia: 27.3	
Castroagudín et al. *Liver Transpl*. 2009; 15: 1792–7 [94]			Before and after conversion: triglyceride: 144.6 ± 88.9 versus 188.8 ± 121.3 mg/dL (*P* = 0.121); cholesterol: 192.8 ± 34.8 versus 241.8 ± 95.5 mg/dL (*P* = 0.024)	
De Simone et al. *Transpl Int*. 2009; 22: 279–86 [95]			Hyperlipemia: 42.5	
De Simone et al. *Liver Transpl*. 2009; 15: 1262–9 [96]			Hypercholesterolemia: 13.9 versus 2.7 (*P* = 0.017)	
Vallin et al. *Clin Transplant*. 2011; 25: 660–9 [99]	Incidence of diabetes did not significantly vary after EVR introduction (before versus after: 30 versus 31)	Incidence of arterial hypertension did not significantly vary after EVR introduction (before versus after: 59 versus 53)	Hyperlipidemia: 37	
Saliba et al. *Liver Transpl*. 2011; 17: 905–13 [98]			Hypertriglyceridemia: 14.6 Hypercholesterolemia: 13.3	

BMI: body mass index; CNI: calcineurin inhibitor; EVR: everolimus; ILTS: 2011 Joint International Congress of the International Liver Transplantation Society; NODM: new-onset diabetes mellitus; NS: nonsignificant; SD: standard deviation; SRL: sirolimus; TAC: tacrolimus; TAC-RD: reduced-dose tacrolimus (*C*0: 3–5 ng/mL); TAC-SD: standard-dose tacrolimus (*C*0: 6–10 ng/mL).

*P* values are included where available.

*Treatment group differences with an exploratory *P* value ≤ 0.05.

**Table 6:** The effects of sirolimus in liver transplant recipients with concomitant hepatocellular carcinoma.

	Overall survival rate at 1 year after transplant (%)	Overall survival rate at 2 years after transplant (%)	Overall survival rate at 3 years after transplant (%)	Overall survival rate at 4 years after transplant (%)	Overall survival rate at 5 years after transplant (%)	Mortality risk ratio (SRL : CNI)
Maintenance dosing						
Toso et al. *Hepatology.* 2010; 51: 1237–43 [65]			85.6 versus 79.2 (SRL versus SRL-free)		83.1 versus 68.7 (SRL versus SRL-free, *P* ≤ 0.05)	
Kneteman et al. *Liver Transpl*. 2004; 10: 1301–11 [63]	94.1 (Milan criteria) 90.5 (extended criteria)			81.1 (Milan criteria), 76.8 (extended criteria)		

*De novo* dosing						
Chinnakotla et al. *Liver Transpl*. 2009; 15: 1834–42 [55]	94 versus 79 (SRL versus TAC)		85 versus 66 (SRL versus TAC)		80 versus 59 (SRL versus TAC, *P* = 0.001)	
Zhou et al. *Transplant Proc*. 2008; 40: 3548–53 [59]	90.67 versus 61.60 (SRL versus TAC, *P* = 0.011)	80.59 versus 53.90 (SRL versus TAC, *P* = 0.011)				
Zimmerman et al. *Liver Transpl*. 2008; 14: 633–8 [15]	95.5 versus 83 (SRL versus CNI)				78.8 versus 62 (SRL versus CNI)	0.672 (*P* = 0.087)

Late conversion (>3 months after transplantation)						
Vivarelli et al. *Transplant Proc*. 2010; 42: 2579–84 [80]			84			

*P* values are included where available.

CNI: calcineurin inhibitor; NS: nonsignificant; SRL: sirolimus; TAC: tacrolimus.

**Table 7:** Effects of mTOR inhibitors on hepatitis C virus and fibrosis progression in hepatitis C virus liver transplant recipients.

	Patient survival	Graft survival	Fibrosis	HCV recurrence rate	Other
Sirolimus					

Maintenance dosing					
Wagner et al. *Int Immunopharmacol*. 2010; 10: 990–3 [58]	95% survival in whole group; lower survival in SRL versus CNI according to log rank test (*P* < 0.03)			Viral load (×10^6^): 6.9 versus 21.3 (SRL versus CNI, *P* < 0.001)	

*De novo* dosing					
McKenna et al. *Am J Transplant*. 2011; 11: 2379–87 [57]	1 year after transplant: patient survival: 92.5% versus 87.9% (SRL versus control, *P* = 0.15) 5 years after transplant: similar patient and graft survival (Kaplan–Meier analysis)		Fibrosis state ≥2, 1 year after transplant: 15.3% versus 36.2% (SRL versus control, *P* < 0.0001) 2 years after transplant: 30.1% versus 50.5% (SRL versus control, *P* = 0.001)	Mean HCV RNA, 1 year after transplant: 9.16 × 10^6^ versus 6.90 × 10^6^ (SRL versus control, *P* = NS) 2 years: 6.12 × 10^6^ versus 4.83 × 10^6^ (*P* = NS)	
Asthana et al. *Can J Gastroenterol*. 2011; 25: 28–34 [56]			Change in mean fibrosis score: +1.1 versus −0.52 (CNI versus SRL, *P* = 0.002) Change in mean activity: +0.24 versus −0.25 (CNI versus SRL, *P* = 0.056)	HCV recurrence: 75% versus 69.8% (SRL versus controls, *P* = NS)	
Asthana et al. Presented at AASLD 2011 (Abstract 184) [60]					SVR: 60%, 43%, 29% (SRL versus TAC versus CsA) SRL-based immunosuppression impacted SVR (OR 2.41, 95% CI 1.1, 5.5)

Late conversion (>3 months after transplantation)					
Stein et al. Presented at the American Transplant Congress 2011 (Abstract 817) [79]		1-, 3- and 5-year allograft survival probability (%): 100, 92, 78 (SRL) and 100, 95, 90 (TAC), log rank −0.6, respectively	1- and 3-year fibrosis and activity scores: *P* = NS (SRL versus TAC)		

Everolimus					

*De novo* dosing					
Dopazo et al. Presented at ILTS; 2011 (Abstract P-460) [102]			Fibrosis stages I and II–IV, respectively: 8% and 30% (EVR), and 5% and 36% (control group) (*P* = NS)		

Maintenance dosing					
Grazi et al. Presented at ILTS; 2011 (Abstract P-256) [88]				HCV recurrence rate: 54% versus 33% (EVR versus TAC, *P* = NS)	

Early conversion (≤3 months after transplantation)					
Masetti et al. *Am J Transplant*. 2010; 10: 2252–62 [89]				HCV recurrence rate: 65% versus 75% (EVR versus CsA, *P* = 1)	

*P* values are included where available.

AASLD: The Liver Meeting 62nd Annual Meeting of the American Association for the Study of Liver Diseases; EVR: everolimus; HCV: hepatitis C virus; ILTS: 2011 Joint International Congress of the International Liver Transplantation Society; NS: nonsignificant; SRL: sirolimus; SVR: sustained virologic response; TAC: tacrolimus.

**Table 8:** Other properties of mTOR inhibitors.

mTOR inhibitor	Effect of mTOR inhibitors on *de novo* tumors	
EVR	Bilbao et al. *Transplant Proc*. 2009; 41: 2172–6 [92]	Six recipients with *de novo* tumors received oncological treatment/surgery and were converted to EVR-based IS. Five were disease-free at follow-up (mean 10 ± 9 months after conversion)
EVR	Fischer et al. *Am J Transplant*. 2012; 12: 1855–65 [50]	Neoplasms: benign, malignant and unspecified (including cysts and polyps): 4.0% versus 8.8% (EVR versus CNI, *P* = NS)
SRL	Jiménez-Romero et al. *Hepatogastroenterology*. 2011; 58: 115–21 [62]	11 of 16 recipients who developed *de novo* tumors and were switched from CNI/MMF to SRL monotherapy were alive at follow-up (mean follow-up 15.7 months)
SRL	Vivarelli. *Transplant Proc*. 2010; 42: 2579–84 [80]	3 of 4 recipients with *de novo* tumors and who converted to SRL-based IS were alive and tumor-free up to 33 months after conversion

mTOR inhibitor	Effect of mTOR inhibitors on neurological symptoms	

EVR	Bilbao et al. *Transplant Proc*. 2009; 41: 2172–6 [92]	3/3 cases of neurotoxicity resolved
EVR	Masetti et al. *Am J Transplant*. 2010; 10: 2252–62 [89]	Minor neurological complications: EVR: 3/52 (5.8%), CsA: 5/26 (19.2%) (*P* = 0.11)
SRL	Forgacs et al. *Transplant Proc*. 2005; 37: 1912–4 [70]	7/7 recipients showed improvement or resolution of neurological symptoms
SRL	Maramattom and Wijdicks*Neurology*. 2004; 63: 1958–9 [64]	No neurotoxicity in 52 recipients treated with SRL from 2001–04
SRL	Morard et al.* Liver Transpl*. 2007; 13: 658–64 [75]	Severe CNI-associated neurological symptoms improved in 6/6 recipients who converted from CNI
SRL	Vivarelli. *Transplant Proc*. 2010; 42: 2579–84 [80]	Complete resolution of neurological symptoms in 14/16 recipients who converted from CNI

*P* values are included where available.

CNI: calcineurin inhibitor; CsA: cyclosporine A; EVR: everolimus; HCV: hepatitis C virus; IS: immunosuppression; MMF: mycophenolate mofetil; SRL: sirolimus; TAC: tacrolimus.

## Abbreviations

BPAR: Biopsy-proven acute rejection
CMV: Cytomegalovirus
CNI: Calcineurin inhibitor
EMA: European Medicines Agency
EVR: Everolimus
FDA: US Food and Drug Administration
GFR: Glomerular filtration rate
HAT: Hepatic artery thrombosis
HCC: Hepatocellular carcinoma
HCV: Hepatitis C virus
MDRD: Modification of diet in renal disease
MMF: Mycophenolate mofetil
mTOR: Mammalian target of rapamycin
NODM: New-onset diabetes mellitus
SRL: Sirolimus
SRTR: Scientific Registry of Transplant Recipients
TAC-RD: Reduced-exposure tacrolimus.
